# Tumor enucleation versus conventional partial nephrectomy for localized renal tumors: a systematic review and meta-analysis of functional, perioperative, and margin outcomes

**DOI:** 10.3389/fonc.2026.1853974

**Published:** 2026-06-26

**Authors:** Shijie Jiang, Jingyu Liu, Lu Sun, Yifan Zhu, Ziyi Fan, Shiqing Li, Feng Zhou

**Affiliations:** Department of Urology, the First Affiliated Hospital of Soochow University, Suzhou, Jiangsu, China

**Keywords:** localized renal tumor, major complications, nephron-sparing surgery, positive surgical margins (PSM), renal function, warm ischemia time (WIT)

## Abstract

**Background:**

Tumor enucleation (TE) may preserve more renal parenchyma than conventional partial nephrectomy (PN), but its functional and safety benefits remain debated. This study updated the evidence comparing TE and PN for localized renal tumors, mainly clinical T1 disease.

**Methods:**

PubMed, Embase, Web of Science, the Cochrane Library, Scopus, and CNKI were searched to May 2026. Comparative studies of TE versus PN in adults with localized renal tumors were included. Key outcomes were postoperative eGFR, absolute ΔeGFR, positive surgical margin (PSM), and major complications. Random-effects models were used.

**Results:**

Seventeen studies with 5,249 patients were included. TE was associated with higher postoperative eGFR (MD = 5.64 mL/min/1.73 m², 95% CI 3.64–7.63, P <0.00001; I² = 12%) and smaller absolute ΔeGFR (MD = −2.12 mL/min/1.73 m², 95% CI −3.85 to −0.39, P = 0.02; I² = 14%). TE did not increase PSM risk (OR = 0.73, 95% CI 0.46–1.13, P = 0.16; I² = 25%) and was associated with fewer major complications (OR = 0.48, 95% CI 0.32–0.71, P = 0.0003; I² = 0%). TE also showed shorter WIT, shorter operative time, and lower EBL, but these perioperative outcomes had high heterogeneity. Long-term oncological outcomes could not be pooled.

**Conclusion:**

TE may offer better renal functional preservation and fewer major complications than conventional PN in selected patients with localized renal tumors, without increasing PSM risk. However, evidence for absolute ΔeGFR was limited, perioperative outcomes were heterogeneous, and long-term oncological equivalence remains uncertain.

**Systematic review registration:**

https://www.crd.york.ac.uk/prospero/, identifier PROSPERO CRD420261361330.

## 1 Introduction

Renal cell carcinoma (RCC) is often clinically silent in its early stage and accounts for about 2–3% of adult malignancies worldwide. Its incidence has increased over time, and more than 400,000 new cases are diagnosed each year (1, 2). With the wide use of cross-sectional imaging and ultrasonography, more localized renal tumors are detected at an early stage (3). Because of this, more patients with localized RCC are diagnosed at a stage when cure is still possible (4, 5).

Over the past two decades, partial nephrectomy (PN) has become the standard surgical treatment for localized renal tumors, especially clinical T1 renal tumors, when nephron-sparing surgery is technically feasible (6). The main goals of PN are to achieve negative surgical margins, preserve renal function as much as possible, and reduce perioperative complications. These goals broadly overlap with the concept of the surgical trifecta (7, 8). In conventional PN, a visible rim of normal renal parenchyma is usually removed around the tumor to ensure margin control. However, this approach may cause unnecessary loss of functional nephrons and may impair postoperative renal function preservation (9).

To preserve more renal parenchyma, tumor enucleation (TE) has received increasing attention as a nephron-sparing technique. TE follows the natural plane between the tumor pseudocapsule and the surrounding normal renal parenchyma. Therefore, TE may allow complete tumor removal while preserving more functional renal tissue and reducing unnecessary injury to renal vessels and the collecting system (10–12). Initially, TE was mainly used in imperative nephron-sparing settings, such as solitary kidney, pre-existing renal insufficiency, multifocal renal tumors, and hereditary renal cancer syndromes. In recent years, with the development of minimally invasive and robot-assisted surgery, TE has gradually been extended to selected patients with clinical T1 renal tumors (6, 13–15).

As the use of TE has expanded, its true benefit compared with conventional PN still needs further evaluation. Several systematic reviews and meta-analyses have already compared TE with conventional PN (16–18). Therefore, this clinical question is not entirely new. However, after earlier reviews were published, new comparative studies have become available, including contemporary robot-assisted series and randomized studies. These new data may further affect our understanding of the renal functional benefit, perioperative safety, and margin control of TE. Therefore, the current evidence needs to be updated and reassessed.

In this study, we systematically reviewed the available comparative studies and performed an updated meta-analysis to compare TE with conventional PN in adult patients with localized renal tumors. The included population mainly involved clinical T1 renal tumors. The key outcomes included postoperative eGFR, absolute decline in eGFR (ΔeGFR), positive surgical margin (PSM) rate, and major complications defined as Clavien-Dindo grade ≥III. Secondary outcomes included warm ischemia time (WIT), operative time, and estimated blood loss (EBL). With these outcomes, this study aimed to better assess the differences between TE and conventional PN in renal function preservation, surgical safety, and margin control.

## 2 Methods

### 2.1 Criteria for considering studies

**Figure 1:**
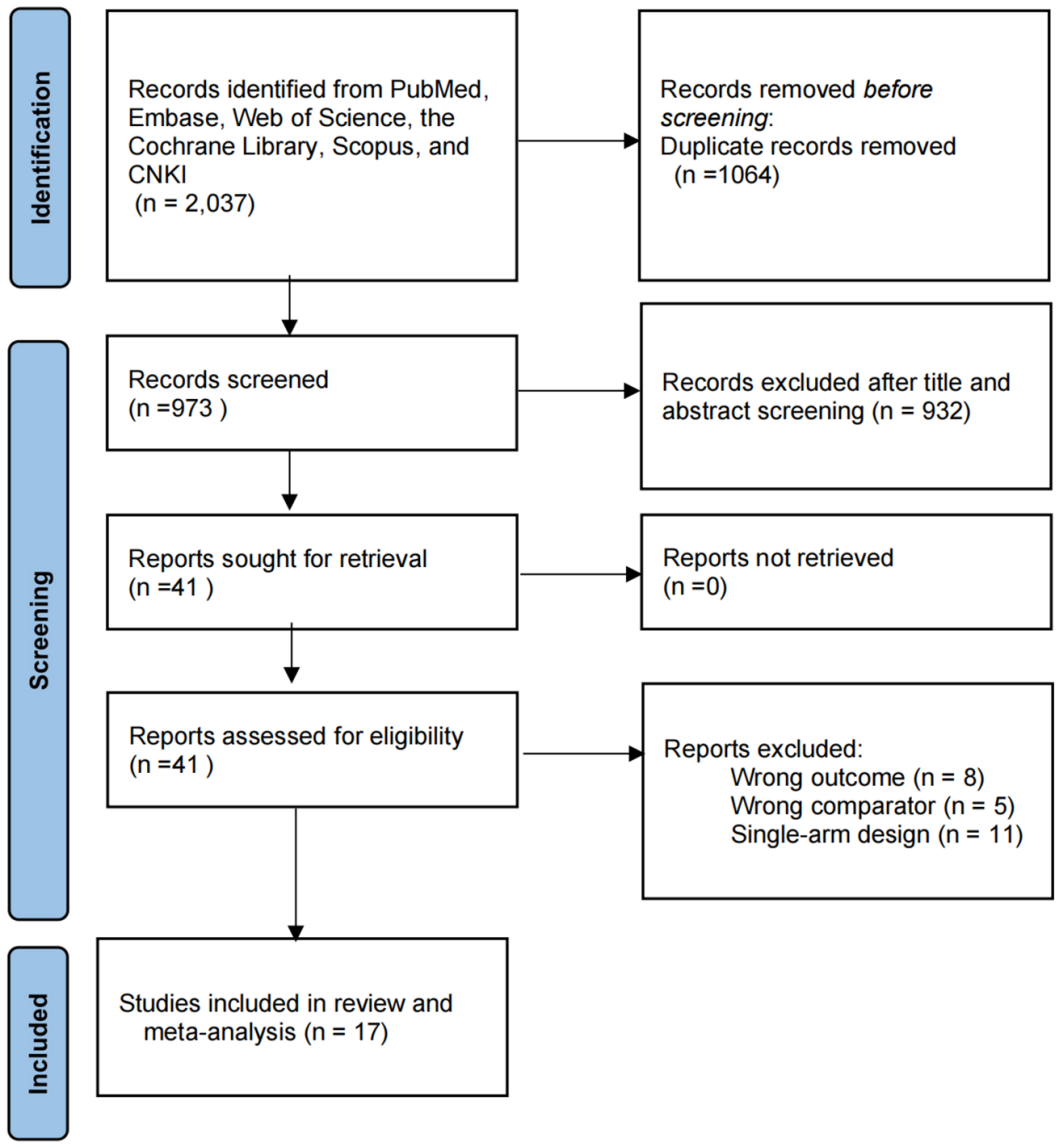
PRISMA flow diagram of study selection.

This review followed the PRISMA 2020 reporting guideline (**Figure 1**) (19). The protocol was prepared before data extraction. It defined the eligibility criteria, outcomes, subgroup analyses, and statistical methods in advance. The protocol was registered prospectively in PROSPERO (CRD420261361330).

### 2.2 Search methods

We searched PubMed, Embase, Web of Science, the Cochrane Library, Scopus, and CNKI. The first search included PubMed, Embase, Web of Science, and the Cochrane Library from database inception to November 2025. Scopus and CNKI were later added to improve coverage, and all databases were updated to May 2026. The search covered terms for renal tumors, tumor enucleation, and partial nephrectomy, including “renal tumor,” “renal cell carcinoma,” “kidney neoplasm,” “tumor enucleation,” “simple enucleation,” “enucleoresection,” “partial nephrectomy,” and “nephron-sparing surgery.” No language limits were used. Full search strategies, including CNKI Chinese terms, are shown in **Supplementary Table 1**. Reference lists of included studies and relevant reviews were checked manually. Clinical trial registries and other gray literature sources were not searched separately.

### 2.3 PICOS

The review question was built using the PICOS framework. The population included adult patients with localized renal tumors, mainly clinical T1 renal tumors. The intervention was tumor enucleation. The comparator was conventional partial nephrectomy. The outcomes of interest included renal functional outcomes, perioperative outcomes, margin-related outcomes, oncological outcomes, and safety outcomes. Eligible study designs were comparative clinical studies, including randomized controlled trials and observational cohort studies.

### 2.4 Inclusion and exclusion criteria

Studies were included if they met the following criteria: (1) clinical studies directly comparing TE with conventional PN; (2) adult patients aged ≥18 years with localized, non-metastatic renal tumors who underwent nephron-sparing surgery; (3) according to the definitions used in the original studies, TE generally referred to tumor removal along the tumor pseudocapsule or the natural tumor-parenchyma plane without intentional removal of a visible rim of normal renal parenchyma, and PN generally referred to standard partial nephrectomy with intentional removal of a rim of normal renal parenchyma around the tumor. Because the detailed operative descriptions were not fully identical across studies, a study was considered eligible when the original authors clearly defined the two groups as TE and conventional PN and when the outcomes of the two groups were directly comparable; (4) at least one renal functional, perioperative, margin-related, oncological, or safety outcome was reported; and (5) data could be extracted or calculated. Studies that mainly included localized, non-metastatic renal tumors were also considered eligible when TE and PN outcomes were directly comparable.

The exclusion criteria were as follows: (1) reviews, case reports, conference abstracts, and other non-original publications; (2) non-comparative studies or studies without comparable outcome data; and (3) duplicate publications or studies with overlapping cohorts. For overlapping cohorts, the report with the largest sample size or the most complete dataset was retained.

### 2.5 Data extraction and outcomes

After duplicates were removed, two reviewers (J.S. and L.J.) screened titles and abstracts in Rayyan. They then assessed the full texts of potentially eligible studies. Data were extracted with a prespecified Excel sheet by the same two reviewers. Disagreements were resolved by discussion, and L.S. made the final decision when needed. Inter-reviewer agreement was not formally calculated.

Outcomes were grouped as renal functional outcomes, perioperative outcomes, margin-related/oncological outcomes, and safety outcomes. The key outcomes were postoperative eGFR, absolute ΔeGFR, PSM rate, and major complications. Major complications were defined as Clavien-Dindo grade ≥III (20). Studies that reported only overall complications without clear grading were described but were not included in the pooled analysis of major complications.

Secondary outcomes were WIT, operative time, and EBL. When available, long-term oncological outcomes were also collected, including local recurrence, distant metastasis, recurrence-related progression, cancer-specific survival, and overall survival. These outcomes were summarized descriptively. We also extracted postoperative bleeding and urine leakage when they were reported separately. Because the definitions and reporting methods were inconsistent, these events were not pooled as prespecified outcomes.

Because the detailed operative definitions of TE and PN were not fully identical across studies, we also extracted the surgical definitions and ischemia management strategies reported in each study. According to the operative descriptions in the original studies, the technical definitions of TE and PN were classified into pseudocapsule-plane/simple enucleation, ablation-assisted zero-ischemia enucleation, Surface-Intermediate-Base (SIB) score-based classification, and modified enucleation techniques. Ischemia management strategies were also classified as complete zero ischemia, partial no-clamping use, surgeon-selected ischemia strategy, and not clearly reported. These technical classifications are summarized in **Supplementary Table 2** and were considered when interpreting clinical heterogeneity.

### 2.6 Quality assessment

The quality of each included study was evaluated by two reviewers (J.S. and L.J.). Any disagreement was discussed first, and L.S. was consulted when no agreement could be reached. For randomized trials, we used the Cochrane Risk of Bias 2 (RoB 2) tool (21). This assessment covered bias from randomization, adherence to assigned interventions, missing outcome data, outcome measurement, and selective outcome reporting. For non-randomized studies, we used the Newcastle-Ottawa Scale. This scale has a total score of 9, and studies scoring 7 or higher were regarded as having good methodological quality. The quality results were used to help interpret the evidence, but they did not determine study exclusion. The detailed domain-level assessments are provided in **Supplementary Table 3**.

### 2.7 Statistical analysis

All analyses were carried out with Review Manager 5.4 and R 4.5.3. For continuous variables, treatment effects were reported as mean differences with 95% confidence intervals. For dichotomous variables, odds ratios with 95% confidence intervals were calculated. If a study reported continuous data as medians with ranges or interquartile ranges, these values were estimated as means and standard deviations by published conversion methods when the distribution appeared suitable (22, 23). If a study provided both unmatched and matched results, the matched cohort data were used in the meta-analysis.

For postoperative eGFR, we only pooled studies that provided absolute postoperative values. These values were either directly available or could be estimated from median-based data by standard methods. Studies reporting only preservation rates, decline rates, or other non-absolute postoperative renal function measures were not pooled for this outcome. This approach was used to avoid excessive indirect estimation.

For ΔeGFR, the pooled analysis was limited to studies that reported absolute changes in eGFR as means and standard deviations. Studies that used percentage decline, change rate, or preservation rate were not combined with absolute ΔeGFR because these measures were not on the same scale. These studies were reviewed descriptively.

Between-study heterogeneity was assessed using the chi-square test and the I² statistic. Because clinical heterogeneity was expected across the included studies in terms of surgical platform, operative definition, ischemia strategy, tumor complexity, surgeon experience, and institutional learning curve, random-effects models were used for all main pooled analyses. An I² value >50% or a chi-square test P value <0.10 was considered to indicate substantial statistical heterogeneity. All statistical tests were two-sided, and P <0.05 was considered statistically significant. When at least 10 studies were available for an outcome, publication bias was assessed by visual inspection of funnel plots. For outcomes with fewer than 10 studies, formal publication bias assessment was not performed because funnel plots are unstable in this setting.

### 2.8 Subgroup, meta-regression, and sensitivity analyses

Robustness was examined by leave-one-out analysis. Each study was removed once, and the pooled estimate was recalculated. Changes in effect direction, statistical significance, and heterogeneity were then recorded.

When enough studies were available, subgroup analyses were performed by surgical platform and study design. Surgical platform was grouped as robot-assisted surgery or non-robotic surgery. Study design was grouped as randomized controlled trial or observational study. These analyses were used to examine whether the pooled results were consistent across subgroups and whether heterogeneity decreased within subgroups.

For WIT, operative time, and EBL, which showed high heterogeneity, exploratory meta-regression was performed when the number of studies was sufficient. The tested study-level variables included surgical platform, study design, publication year, sample size, and mean tumor size. Tumor complexity score, ischemia strategy, and TE/PN technical definition were also examined when this information was reported. These analyses were considered exploratory because the number of studies was limited and some variables were not fully reported. Therefore, they were used only to help explain heterogeneity and not to infer causal effects.

## 3 Results

### 3.1 Study selection

The database search identified 2,037 records. After duplicate removal, 973 records remained for title and abstract screening. Of these, 932 records were excluded because they did not meet the eligibility criteria. Forty-one reports were sought for retrieval, and all were retrieved. After full-text assessment, 24 reports were excluded: 8 for wrong outcomes, 5 for wrong comparators, and 11 for single-arm design. Finally, 17 studies were included in the systematic review and meta-analysis (**Figure 1**).

### 3.2 Study characteristics

A total of 17 comparative studies published between 2014 and 2024 were included, with 5,249 patients in total (**Table 1**). There were 2,613 patients in the TE group and 2,636 patients in the PN group. 3 studies were prospective randomized controlled trials, and 14 studies were observational comparative studies. Among the observational studies, four used matching or propensity score matching to reduce baseline differences between the TE and PN groups.

**Table 1:** Basic characteristics of the included studies.

Study	Country	Design	Population	Approach	TE/PN, n	Tumor size	Follow-up	Quality
Huang 2016 (24)	China	Randomized controlled trial	cT1a renal tumors	Laparoscopic	44/45	2.65 (1.2–4.0)/3.0 (1.5–4.0) cm*	Median 18 months; ≥12 months	Some concerns
Lu 2023 (25)	China	Randomized controlled trial	Low-/intermediate-complexity cT1 renal tumors (RENAL score <10)	Robot-assisted	190/190	3.5 (2.5–4.0)/3.5 (2.5–4.5) cm*	Median 18 months	Low risk
Wu 2020 (26)	China	Randomized controlled trial	cT1a renal tumors	Laparoscopic	90/93	3.0 (1.0–4.0)/3.0 (1.5–4.0) cm*	Median 24 months; ≥12 months	Some concerns
Longo 2014 (27)	Italy	Propensity score-matched cohort	Clinical T1 renal masses	Open/laparoscopic	198/198	3.0 ± 1.2/3.0 ± 1.4 cm	NR	NOS 8
Minoda 2021 (28)	Japan	Propensity score-matched cohort	Completely endophytic renal tumors	Robot-assisted	45/45	2.7 ± 1.1/2.6 ± 1.3 cm	6–12 months	NOS 8
Takagi 2017 (29)	Japan	Propensity score-matched cohort	Localized renal tumors	Robot-assisted	45/45	3.3 ± 1.0/3.2 ± 1.1 cm	NR	NOS 8
Culpan 2021 (30)	Turkey	Retrospective multicenter cohort	Localized renal tumors	Mixed	848/222	3.56 ± 1.34/3.48 ± 1.39 cm	At discharge (renal function); long-term NR	NOS 8
Dobrota 2020 (31)	Romania	Prospective cohort	cT1 renal masses	3D laparoscopic	29/54	3.4 ± 1.1 cm overall	12 months	NOS 7
Blackwell 2016 (32)	USA	Retrospective cohort	Localized renal tumors	Robot-assisted	57/53	3.0(2.1-3.6)/2.5 (2.2-3.5)	Median 7.2 months	NOS 7
Deng 2015 (33)	China	Retrospective cohort	Localized renal tumors	Laparoscopic	94/85	3.0 ± 0.9/3.2 ± 0.9 cm	NR	NOS 7
Dong 2017 (34)	USA	Retrospective cohort	Localized renal tumors	Mixed	71/373	3.0 (2.1–3.8)/3.3 (2.3–4.5) cm*	3–12 months	NOS 8
Ellis 2024 (35)	USA	Retrospective cohort	Localized renal tumors	Robot-assisted	168/252	2.8 ± 1.2/3.1 ± 1.4 cm	12 months for eGFR; imaging ≤6 months	NOS 8
Lei 2023 (36)	China	Retrospective cohort	Intermediate/high-complexity localized RCC	Robot-assisted	135/224	4.83 ± 0.96/4.60 ± 0.96 cm	Median NR; schedule reported	NOS 7
Lu 2017 (37)	China	Retrospective cohort	Localized RCC	Laparoscopic	280/105	3.7 ± 1.4/3.6 ± 1.6 cm	Median 18 months	NOS 7
Lu 2019 (38)	China	Retrospective cohort	Highly complex renal tumors (PADUA ≥10)	Robot-assisted	94/72	4.66 ± 1.14/4.77 ± 1.03 cm	Median 36–37 months	NOS 7
Mukkamala 2014 (39)	USA	Retrospective cohort	Nonfamilial unifocal renal tumors	Mixed	86/516	2.9 ± 1.6/2.9 ± 1.4 cm	Median 3 years (functional subset)	NOS 7
Zhao 2021 (40)	China	Retrospective cohort	Clinical T1b RCC	Robot-assisted	139/64	4.5 ± 1.0/4.8 ± 1.1 cm	Median 30–32 months	NOS 7

TE, tumor enucleation; PN, partial nephrectomy; NOS, Newcastle-Ottawa Scale; RCC, renal cell carcinoma; SIB, Surface-Intermediate-Base; NR, not reported. * indicates median with range or interquartile range. For matched studies, matched cohort data were used when available. Detailed TE/PN technical definitions and ischemia strategies are summarized in **Supplementary Table 2**.

The included studies were conducted in China, Japan, the United States, Italy, Turkey, and Romania. The study populations mainly consisted of adult patients with localized renal tumors who underwent nephron-sparing surgery. Most cohorts focused on clinical T1 renal tumors, while some studies included broader localized or non-metastatic renal tumor populations, including pT1–2N0M0 disease, localized RCC, localized renal masses, or high-complexity renal tumors. Across studies, the reported mean or median tumor diameter was about 2.6–4.8 cm.

Surgical approaches and technical definitions varied across the included studies. Surgical platforms included robot-assisted, laparoscopic, 3D laparoscopic, open, and mixed approaches. Most studies defined TE as enucleation along the pseudocapsule plane or the natural tumor-parenchyma plane and used standard PN or sharp excision as the comparator. Some studies used ablation-assisted zero-ischemia enucleation, SIB score-based classification, or modified enucleation techniques. Ischemia management strategies were also not fully consistent and included main renal artery clamping, selective clamping, no-clamping, and zero-ischemia strategies. The related classifications are shown in **Supplementary Table 2**.

Some studies used matching methods, including propensity score matching, to improve comparability between TE and PN groups. Matched cohort data were used when available. Median-based continuous data were converted to estimated means and standard deviations using established methods.

### 3.3 Quality assessment and risk of bias

Among the 17 included studies, 14 were non-randomized comparative studies and were assessed using the Newcastle-Ottawa Scale. NOS scores ranged from 7 to 8, suggesting generally acceptable methodological quality. However, because most studies were observational, residual confounding and selection bias could not be excluded. The three randomized controlled trials were assessed using the Cochrane RoB 2 tool. Lu 2023 was judged to have a low risk of bias, while Huang 2016 and Wu 2020 raised some concerns. The full quality assessment results are shown in **Supplementary Table 3**.

### 3.4 Meta-analysis results

#### 3.4.1 Renal functional outcomes

##### 3.4.1.1 Postoperative eGFR

**Figure 2:**
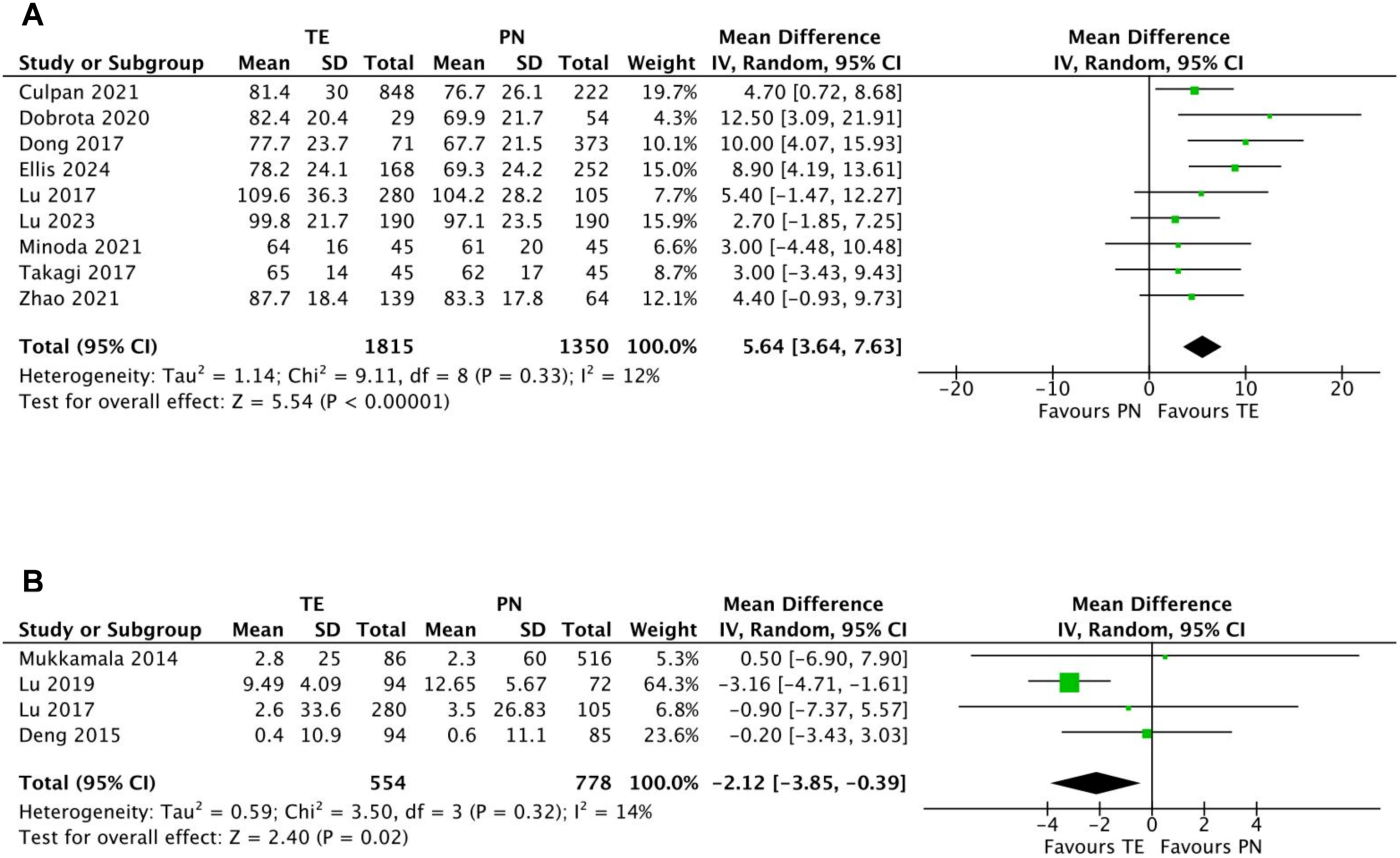
Forest plots of renal functional outcomes. **(A)** postoperative eGFR; **(B)** Absolute ΔeGFR.

Nine studies were included in the analysis of postoperative eGFR, with 1,815 patients in the TE group and 1,350 patients in the PN group (**Figure 2A**). According to the prespecified analytic strategy, only studies with directly extractable absolute postoperative eGFR values or data that could be converted from medians using standard methods were included. Studies reporting only functional preservation rates, decline rates, or other non-comparable formats were not included in this pooled analysis.

The pooled estimate favored TE for postoperative eGFR (MD = 5.64 mL/min/1.73 m², 95% CI 3.64–7.63, P <0.00001), with low between-study heterogeneity (I² = 12%).

##### 3.4.1.2 eGFR decline (ΔeGFR)

Four studies reported absolute ΔeGFR in a format suitable for pooling, including 554 TE patients and 778 PN patients (**Figure 2B**). TE showed a smaller eGFR decline than PN (MD = −2.12 mL/min/1.73 m², 95% CI −3.85 to −0.39, P = 0.02), with low heterogeneity (I² = 14%).

**Figure 3:**
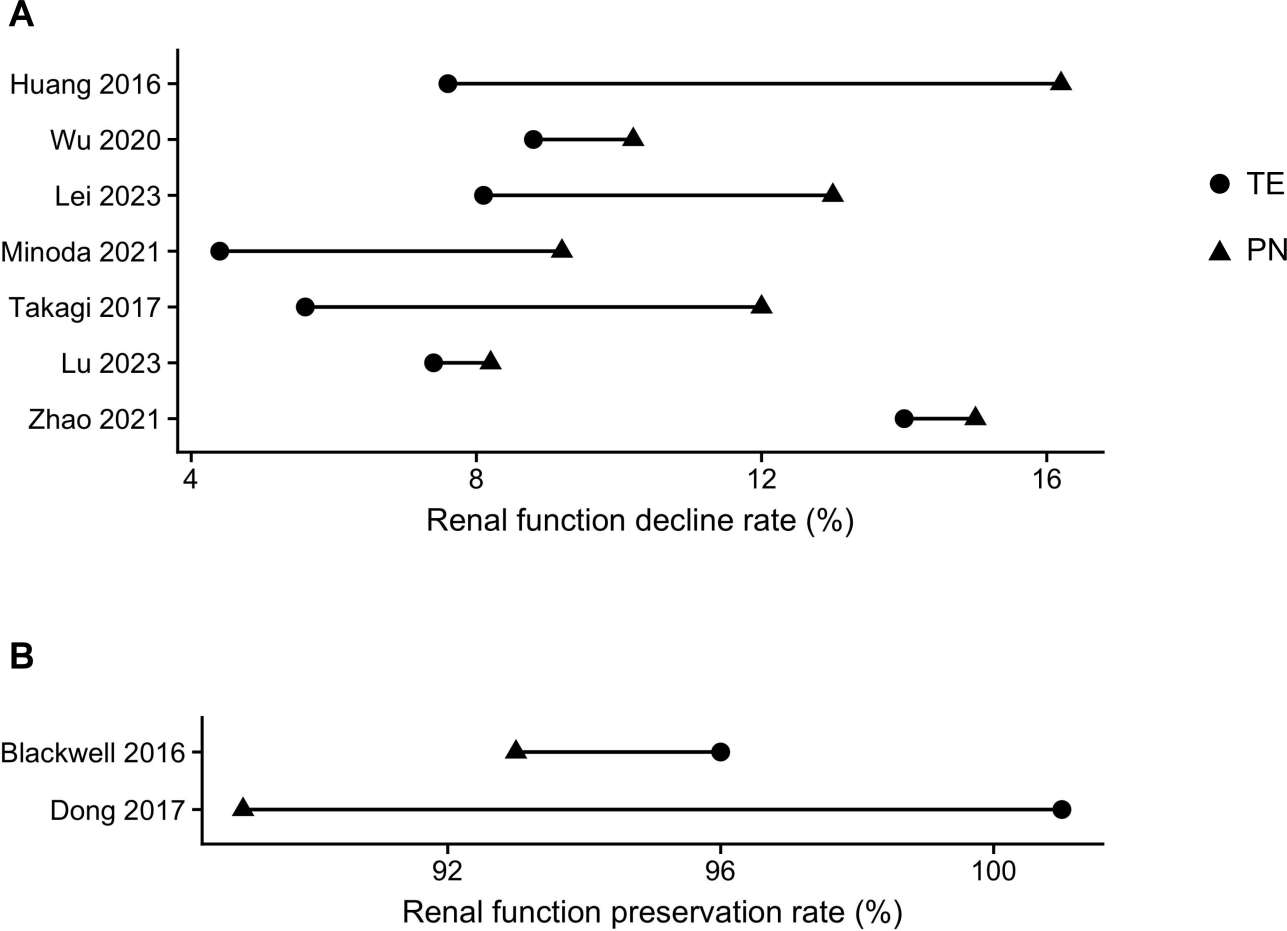
Descriptive summary of renal function changes reported in non-poolable formats. **(A)** Renal function decline rate. **(B)** Renal function preservation rate.

Some other studies reported renal functional change as percentage decline, change rate, or preservation rate. Because these measures had different units and definitions, they were not combined with absolute ΔeGFR and were instead presented descriptively. Overall, most studies showed a lower renal function decline rate or a higher renal function preservation rate in the TE group, which was directionally consistent with the absolute ΔeGFR analysis (**Figure 3**).

#### 3.4.2 Margin-related and safety outcomes

##### 3.4.2.1 Positive surgical margin

**Figure 4:**
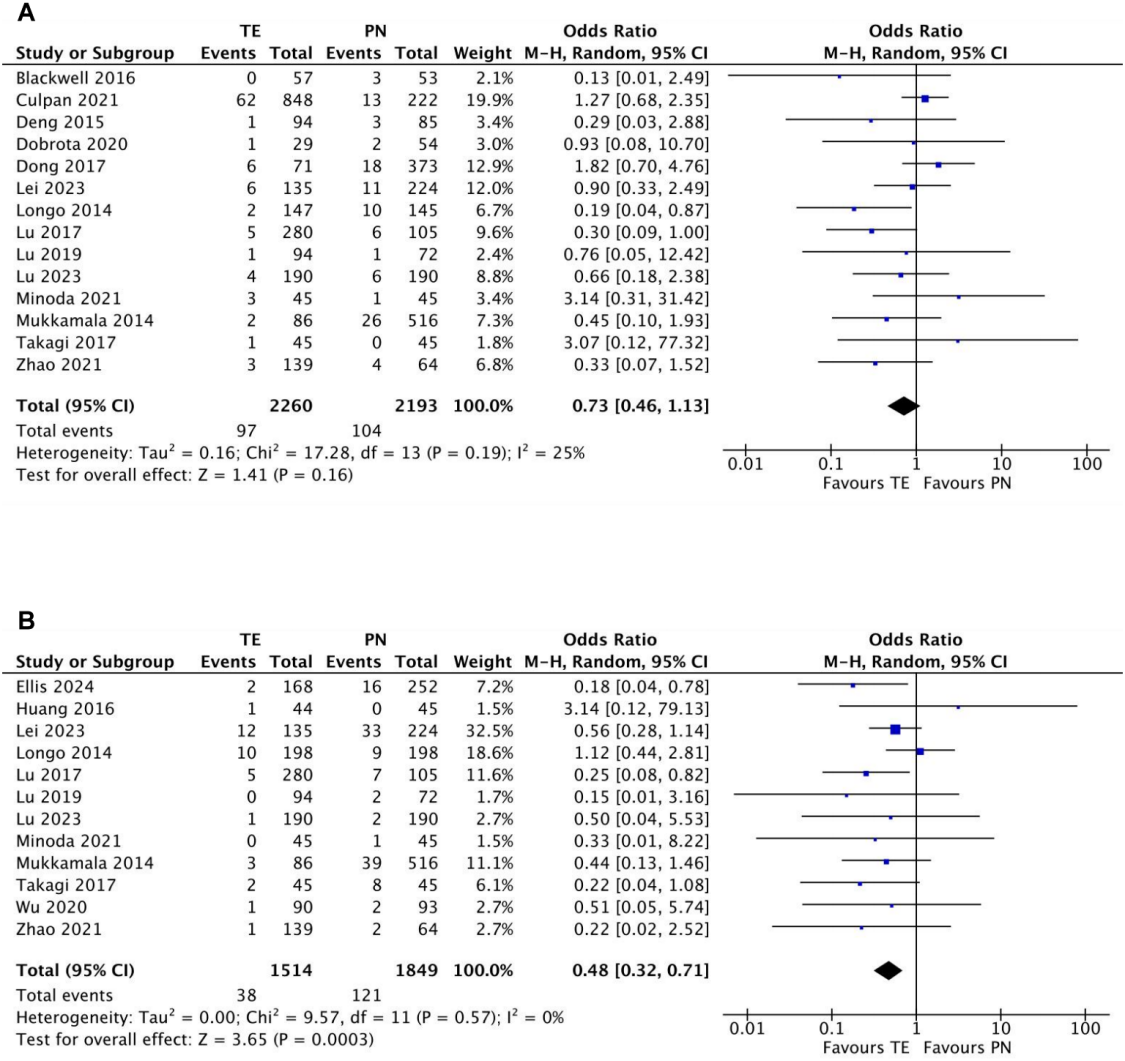
Forest plots of oncologic and safety outcomes. **(A)** positive surgical margin; **(B)** major complications.

Fourteen studies reported positive surgical margin rates, with 2,260 TE cases and 2,193 PN cases (**Figure 4A**). No significant difference in PSM risk was observed between TE and PN (OR = 0.73, 95% CI 0.46–1.13, P = 0.16), with low heterogeneity (I² = 25%). Therefore, TE did not show a higher PSM risk than PN. It should be noted that PSM is only an oncological surrogate endpoint and cannot prove long-term oncological equivalence between the two techniques.

##### 3.4.2.2 Major complications

Twelve studies were included in the analysis of major complications, defined as Clavien-Dindo grade ≥III, with 1,514 patients in the TE group and 1,849 patients in the PN group (**Figure 4B**). The pooled analysis showed that the risk of major complications was lower in the TE group than in the PN group (OR = 0.48, 95% CI 0.32–0.71, P = 0.0003), with no observed heterogeneity (I² = 0%).

##### 3.4.2.3 Long-term oncological outcomes

Long-term oncological outcomes were reported in different ways across the included studies. Time-to-event data, such as hazard ratios for recurrence-free survival, cancer-specific survival, or overall survival, were not available. Therefore, long-term oncological outcomes could not be pooled. The available studies reported few recurrence, metastasis, or cancer-specific death events during follow-up. However, differences in follow-up duration and reporting format limited direct comparison between studies. Therefore, the current pooled evidence could not confirm the long-term oncological durability of TE.

#### 3.4.3 Perioperative outcomes

##### 3.4.3.1 Warm ischemia time

**Figure 5:**
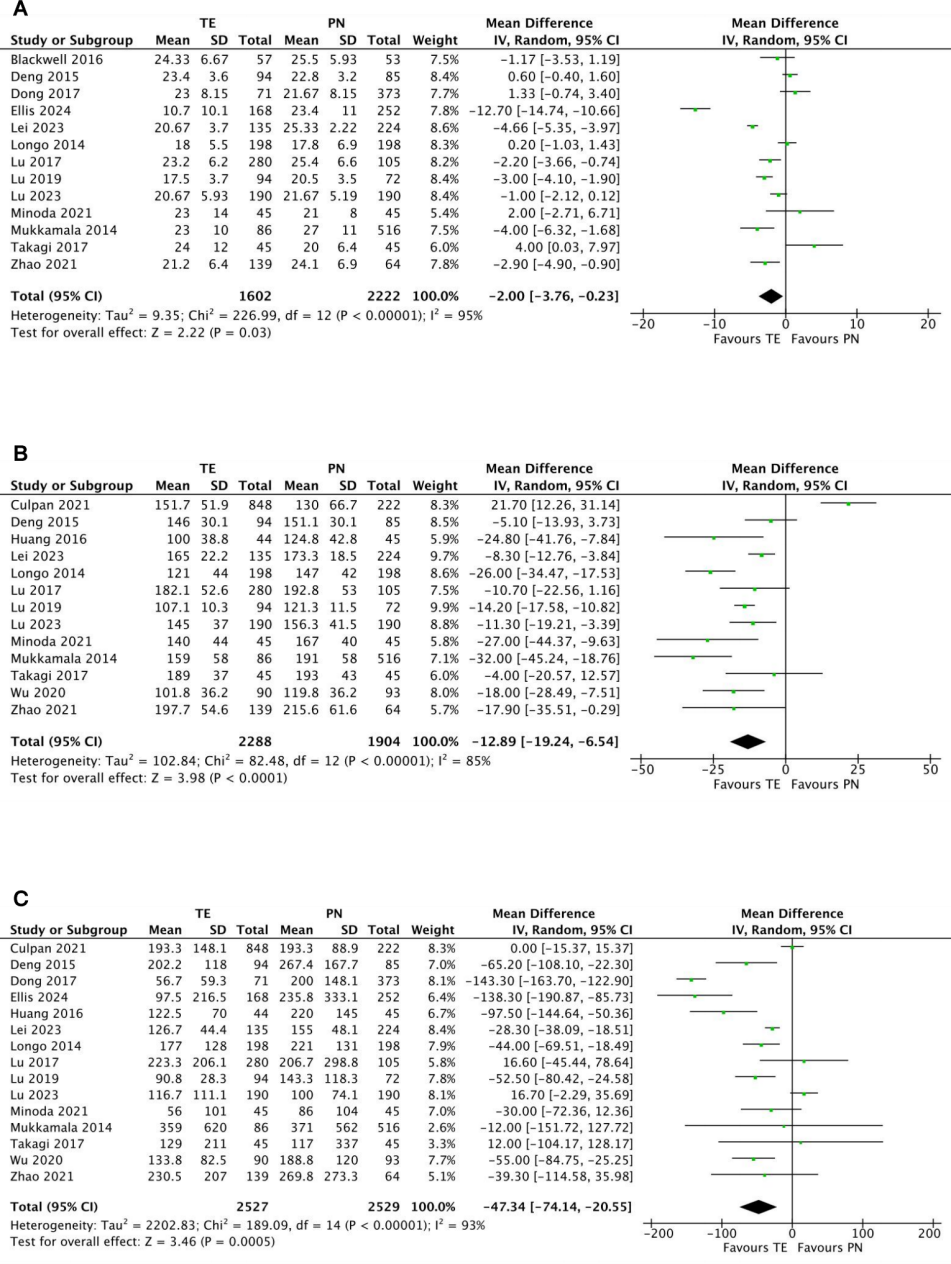
Forest plots of perioperative outcomes. **(A)** warm ischemia time; **(B)** operative time; **(C)** estimated blood loss.

Thirteen studies were included in the WIT analysis, with 1,602 patients in the TE group and 2,222 patients in the PN group (**Figure 5A**). WIT was shorter in the TE group than in the PN group (MD = −2.00 min, 95% CI −3.76 to −0.23, P = 0.03), but heterogeneity was high (I² = 95%). This may reflect differences in ischemia management across studies, so the pooled WIT result should be interpreted with caution.

##### 3.4.3.2 Operative time

Thirteen studies were included in the analysis of operative time, with 2,288 patients in the TE group and 1,904 patients in the PN group (**Figure 5B**). Operative time was shorter in the TE group than in the PN group (MD = −12.89 min, 95% CI −19.24 to −6.54, P <0.0001), but heterogeneity was high (I² = 85%). This variation may reflect differences in case mix and operative practice.

##### 3.4.3.3 Estimated blood loss

Fifteen studies were included in the analysis of estimated blood loss, with 2,527 patients in the TE group and 2,529 patients in the PN group (**Figure 5C**). Estimated blood loss was lower in the TE group than in the PN group (MD = −47.34 mL, 95% CI −74.14 to −20.55, P = 0.0005), but heterogeneity was high (I² = 93%). Therefore, this result should be interpreted as a general trend favoring TE rather than as a precise estimate of blood loss reduction.

### 3.5 Subgroup analysis and meta-regression

We performed subgroup analyses according to study design and surgical platform. Study design was divided into randomized controlled trials and observational studies. Surgical platform was divided into robot-assisted surgery and non-robotic/mixed surgical approaches. These analyses were mainly used to assess whether study design or surgical platform might affect the pooled effect between TE and PN. Because only a few studies directly reported absolute ΔeGFR and all of them were non-randomized studies, subgroup analysis by randomized controlled trial versus observational study was not performed for ΔeGFR.

When studies were grouped by study design, the main findings were broadly consistent across randomized and observational studies. For postoperative eGFR, only one randomized trial was available, and this subgroup did not show a significant difference between TE and PN (MD = 1.60, 95% CI −2.95 to 6.15, P = 0.49). In contrast, observational studies showed higher postoperative eGFR after TE (MD = 6.02, 95% CI 3.42 to 8.63, P <0.00001), but the subgroup difference was not statistically significant (P = 0.10). For PSM, neither subgroup showed a significant difference between TE and PN, and there was no evidence of a subgroup effect (P = 0.89). For major complications, heterogeneity was low in both randomized trials and observational studies (I² = 0% and 3%, respectively). Overall, study design did not clearly modify the comparison between TE and PN, although the small number of randomized trials limited the certainty of these subgroup findings.

After stratification by surgical platform, the directions of effect for most outcomes were also generally consistent. For absolute ΔeGFR, only exploratory subgroup analysis by surgical platform was performed. The effect directions in both the robot-assisted subgroup and the non-robotic/mixed surgery subgroup favored TE. However, the robot-assisted subgroup included only one study, so this result should be interpreted as descriptive and exploratory. For PSM, neither the robot-assisted subgroup nor the non-robotic/mixed surgery subgroup showed an increased PSM risk with TE. For major complications, both surgical platform subgroups showed a lower risk in the TE group, and the test for subgroup differences was not significant (P = 0.87). In the robotic subgroup, heterogeneity was low for PSM (I² = 0%), and heterogeneity was low to moderate for postoperative eGFR and operative time (I² = 28% and I² = 41%, respectively). Overall, the tests for subgroup differences did not show a clear modifying effect of surgical platform. Therefore, surgical platform may not be a major source of heterogeneity. However, because some subgroups included few studies, these results should still be interpreted with caution.

Because WIT, operative time, and EBL showed high heterogeneity in the overall analysis, we further performed exploratory univariable meta-regression for these outcomes. The study-level covariates included publication year, sample size, mean tumor size, surgical platform, study design, TE technique type, ischemia strategy, and tumor complexity. Overall, these variables did not consistently explain between-study heterogeneity in WIT, operative time, or EBL. Mean tumor size was not significantly associated with the effect sizes for WIT, operative time, or EBL (P = 0.531, 0.357, and 0.370, respectively). Surgical platform and tumor complexity also did not show a consistent modifying effect across the three perioperative outcomes. Because the number of included studies was limited and because tumor complexity, surgeon experience, center volume, and detailed technical definitions of TE and PN were not sufficiently reported across studies, these meta-regression results should be interpreted with caution. Detailed regression coefficients, 95% CIs, P values, and residual heterogeneity are shown in **Supplementary Table 4**.

### 3.6 Sensitivity analysis

Leave-one-out sensitivity analyses were performed for all pooled outcomes (**Table 2**). Overall, postoperative eGFR, PSM, and major complications were relatively robust, while absolute ΔeGFR and WIT showed more limited stability. For postoperative eGFR, after sequential omission of each study, the pooled MD remained statistically significant, ranging from 5.00 to 6.17.

**Table 2:** Leave-one-out sensitivity analyses of pooled outcomes.

Outcome	Primary pooled estimate	Range after leave-one-out omission	Statistical significance after omission	Heterogeneity after omission	Interpretation
Postoperative eGFR	MD 5.64 (95% CI 3.64 to 7.63)	MD 5.00 to 6.17	Always significant	Not materially changed	Robust
ΔeGFR	MD -2.12 (95% CI -3.85 to -0.39)	MD -2.90 to -0.23	Not always significant	Low heterogeneity; limited by few studies	Directionally consistent but less stable
Positive surgical margin	OR 0.73 (95% CI 0.46 to 1.13)	OR 0.64 to 0.84	Never significant	Not materially changed	Stable null finding
Major complications	OR 0.48 (95% CI 0.32 to 0.71)	OR 0.39 to 0.52	Always significant	I² = 0% throughout	Highly robust
Warm ischemia time	MD -2.00 (95% CI -3.76 to -0.23)	MD -1.18 to -2.38	Not always significant	Persistent heterogeneity	Directionally consistent but less stable
Operative time	MD -12.89 (95% CI -19.24 to -6.54)	MD -11.40 to -15.38	Always significant	Persistent heterogeneity	Directionally robust, with persistent heterogeneity
Estimated blood loss	MD -47.34 (95% CI -74.14 to -20.55)	MD -37.98 to -53.08	Always significant	Persistent heterogeneity	Directionally robust, with persistent heterogeneity

eGFR, estimated glomerular filtration rate; ΔeGFR, decline in estimated glomerular filtration rate; MD, mean difference; OR, odds ratio; CI, confidence interval; I², inconsistency index.

For ΔeGFR, the main analysis showed a smaller decline in eGFR in the TE group. However, because only four studies directly reported combinable absolute change values, leave-one-out analysis showed limited stability. After sequential omission of each study, the pooled MD ranged from −2.90 to −0.23. The direction of effect generally still favored TE, but after omission of some studies, the pooled result was no longer statistically significant.

The PSM result was also stable. After sequential omission of each study, the pooled OR remained non-significant, ranging from 0.64 to 0.84. The result for major complications was relatively robust. The pooled OR ranged from 0.39 to 0.52, and I² remained 0% throughout.

For perioperative outcomes, the effect direction after leave-one-out analysis generally still favored TE, but stability was weaker. Operative time and EBL remained statistically significant after sequential omission of studies, but heterogeneity remained. In contrast, WIT showed weaker stability. Although the effect direction generally still favored TE, after omission of some studies, the pooled result was no longer statistically significant. This suggests that the WIT result may be more easily affected by individual studies and between-study differences.

### 3.7 Publication bias assessment

Funnel plots were inspected for outcomes with at least 10 studies and suitable data (**Supplementary Figure 4**). This assessment was exploratory. No clear asymmetry was observed for PSM or major complications. Some asymmetry was noted for WIT, operative time, and EBL. Because these perioperative outcomes had high heterogeneity, the asymmetry may reflect differences in study design and surgical practice rather than publication bias alone.

## 4 Discussion

This updated systematic review and meta-analysis showed that TE was associated with better renal function preservation and fewer major complications than conventional PN, without a higher risk of positive surgical margins. Postoperative eGFR was higher in the TE group. The absolute ΔeGFR analysis and the descriptive results based on change rates or preservation rates also supported a smaller decline in renal function after TE. For patients with localized renal tumors mainly involving clinical T1 disease, these findings suggest that TE may provide a better balance between nephron preservation and surgical safety when patients are properly selected.

The comparison between TE and PN is not a new clinical question. Several previous systematic reviews and meta-analyses have already assessed this topic (16–18). The main value of this study is that it updates the available evidence and makes the interpretation more focused. We included recent comparative studies, including newer robot-assisted series and randomized data. We also used stricter rules for renal functional outcomes. Absolute ΔeGFR was analyzed separately from percentage decline, change rate, and preservation rate, because these measures have different units and definitions. We also interpreted PSM as a margin-related endpoint, rather than as proof of long-term oncological equivalence. These choices make this study more focused on the clinical balance between renal function, safety, and margin control.

The possible renal functional benefit of TE may be explained by its anatomical and technical features. TE follows the natural plane between the tumor pseudocapsule and normal renal parenchyma (41). This may reduce removal of normal renal parenchyma and preserve functional nephrons (42, 43). TE may also reduce reconstruction-related injury. Conventional PN usually removes a rim of normal renal parenchyma and may require deeper parenchymal suturing for hemostasis and collecting system closure. This may cause local compression, microvascular injury, and loss of function in part of the remaining renal tissue (2, 44, 45). In suitable cases, TE may reduce deep suturing and parenchymal compression, and this may help preserve vascularized nephron mass.

For renal functional outcomes, this study used ΔeGFR as one of the main functional endpoints. Postoperative eGFR reflects renal function at a given follow-up time, but it can be affected by baseline renal function, age, and comorbidities. In contrast, ΔeGFR better reflects the change in renal function before and after surgery and is less dependent on baseline renal function than postoperative eGFR alone. Therefore, when different nephron-sparing techniques are compared, ΔeGFR may better reflect surgery-related renal functional preservation (44–46). In this study, postoperative eGFR and absolute ΔeGFR both favored TE. However, only four studies directly reported combinable absolute ΔeGFR, so this result should be interpreted as supportive evidence. Other studies reported renal functional change as percentage decline, change rate, or preservation rate. These data were not pooled with absolute ΔeGFR and were summarized descriptively. The overall direction still supported better renal function preservation with TE.

Perioperative outcomes should be interpreted with caution. TE was associated with shorter WIT, shorter operative time, and lower EBL, but all three outcomes showed high heterogeneity. This is expected in surgical meta-analyses, because these outcomes are strongly influenced by real-world surgical factors, such as case selection, operative approach, reconstruction technique, and institutional experience (47–49).

The included studies also differed in TE/PN technical definitions and ischemia strategies. These differences may explain part of the heterogeneity. Subgroup analyses and meta-regression did not show that study design, surgical platform, tumor size, TE technique type, ischemia strategy, or tumor complexity consistently explained the heterogeneity. Therefore, the perioperative results should be viewed as general trends favoring TE, rather than precise estimates of effect size.

For oncological safety, this study did not find a higher PSM risk with TE. This suggests that enucleation along the pseudocapsule plane does not necessarily lead to a higher positive margin rate. At the level of margin control, this finding supports the technical feasibility of TE. However, similar PSM rates should not be interpreted as long-term oncological equivalence (50). PSM is an important intermediate endpoint, but it cannot replace recurrence-free survival, cancer-specific survival, or overall survival (51, 52). Long-term oncological outcomes were reported inconsistently, and combinable time-to-event data were not available. Therefore, this study can only show that TE did not increase PSM risk. It cannot prove long-term oncological equivalence between TE and PN.

TE was also associated with a lower risk of major complications. This suggests that a more parenchyma-sparing resection strategy does not necessarily compromise surgical safety. More precise dissection along the pseudocapsule plane may reduce unnecessary parenchymal removal, deep suturing, vascular injury, and collecting system injury (10, 42–44). However, major complications are a combined outcome and cannot show differences in specific complications. We reviewed postoperative bleeding and urine leakage because they are important after nephron-sparing surgery (53–55). But only a few studies reported these events separately, and definitions were inconsistent. Therefore, reliable pooled analysis was not possible. Future studies should report specific complication types in a standardized way.

This study has several limitations. First, most included studies were retrospective comparative cohorts, and only three randomized controlled trials were available. Selection bias and residual confounding may still exist, even in studies using propensity score matching. Second, some continuous outcomes required conversion from medians and ranges or interquartile ranges into means and standard deviations, which may introduce estimation error. Third, patient-level data were not available, so more detailed adjustment for tumor complexity, baseline renal function, ischemia strategy, and surgeon experience was not possible. Fourth, long-term oncological endpoints could not be pooled because they were reported inconsistently. Fifth, high heterogeneity was present in several perioperative outcomes. Finally, although Scopus and CNKI were added in the updated search, clinical trial registries and other gray literature sources were not searched separately, so unpublished or ongoing studies may have been missed.

## 5 Conclusion

This updated systematic review and meta-analysis showed that, in patients with localized renal tumors mainly involving clinical T1 disease, TE was associated with better renal functional preservation and a lower risk of major complications, without an increased risk of positive surgical margins. The results for postoperative eGFR and absolute ΔeGFR generally supported the renal functional benefit of TE, and the descriptive findings based on change rates and preservation rates showed a similar direction. However, only a small number of studies could be pooled for absolute ΔeGFR, and perioperative outcomes such as WIT, operative time, and EBL showed high heterogeneity. Therefore, these findings should be interpreted with caution. Current evidence supports TE as a feasible nephron-sparing strategy in selected patients, but it still cannot confirm long-term oncological equivalence between TE and conventional PN. Future prospective studies with standardized definitions of surgical techniques, consistent reporting of renal functional outcomes and complications, and longer follow-up are still needed.

## Data Availability

The original contributions presented in the study are included in the article/**Supplementary Material**. Further inquiries can be directed to the corresponding authors.

## Supplementary material

Supplementary Figure 1 Leave-one-out sensitivity analyses of pooled outcomes. **(A)** Postoperative eGFR. **(B)** Absolute ΔeGFR. **(C)** Positive surgical margin. **(D)** Major complications. **(E)** Warm ischemia time. **(F)** Operative time. **(G)** Estimated blood loss.

Supplementary Figure 2 Subgroup analyses according to study design. **(A)** Postoperative eGFR. **(B)** Positive surgical margin. **(C)** Major complications. **(D)** Warm ischemia time. **(E)** Operative time. **(F)** Estimated blood loss.

Supplementary Figure 3 Subgroup analyses according to surgical platform. **(A)** Postoperative eGFR. **(B)** Absolute ΔeGFR. **(C)** Positive surgical margin. **(D)** Major complications. **(E)** Warm ischemia time. **(F)** Operative time. **(G)** Estimated blood loss.

Supplementary Figure 4 Funnel plots for publication bias assessment. **(A)** Positive surgical margin. **(B)** Major complications. **(C)** Warm ischemia time. **(D)** Operative time. **(E)** Estimated blood loss.

Supplementary Table 1 Search strategies.

Supplementary Table 2 Technical Definitions of TE/PN and Zero-Ischemia Use.

Supplementary Table 3 Risk of bias assessment for randomized trials and Newcastle-Ottawa Scale assessment for non-randomized studies.

Supplementary Table 4 Univariable meta-regression analyses for perioperative outcomes.

## References

[1] Bray F Laversanne M Sung H Ferlay J Siegel RL Soerjomataram I . Global cancer statistics 2022: GLOBOCAN estimates of incidence and mortality worldwide for 36 cancers in 185 countries. CA A Cancer J Clin. (2024) 74:229–63. doi: 10.3322/caac.21834 38572751

[2] Padala SA Barsouk A Thandra KC Saginala K Mohammed A Vakiti A . Epidemiology of renal cell carcinoma. World J Oncol. (2020) 11:79–87. doi: 10.14740/wjon1279 32494314 PMC7239575

[3] Rossi SH Hsu R Blick C Goh V Nathan P Nicol D . Meta-analysis of the prevalence of renal cancer detected by abdominal ultrasonography. Br J Surg. (2017) 104:648–59. doi: 10.1002/bjs.10523 28407225

[4] Makino T Kadomoto S Izumi K Mizokami A . Epidemiology and prevention of renal cell carcinoma. Cancers. (2022) 14:4059. doi: 10.3390/cancers14164059 36011051 PMC9406474

[5] Hsieh JJ Purdue MP Signoretti S Swanton C Albiges L Schmidinger M . Renal cell carcinoma. Nat Rev Dis Primers. (2017) 3:17009. doi: 10.1038/nrdp.2017.9 28276433 PMC5936048

[6] Ljungberg B Albiges L Abu-Ghanem Y Bedke J Capitanio U Dabestani S . European association of urology guidelines on renal cell carcinoma: the 2022 update. Eur Urol. (2022) 82:399–410. doi: 10.1016/j.eururo.2022.03.006 35346519

[7] Brassetti A Anceschi U Bertolo R Ferriero M Tuderti G Capitanio U . Surgical quality, cancer control and functional preservation: introducing a novel trifecta for robot-assisted partial nephrectomy. Minerva Urol Nefrol. (2020) 72:82–90. doi: 10.23736/S0393-2249.19.03570-7 31833720

[8] Hung AJ Cai J Simmons MN Gill IS . Trifecta” in partial nephrectomy. J Urol. (2013) 189:36–42. doi: 10.1016/j.juro.2012.09.042 23164381

[9] Mir MC Ercole C Takagi T Zhang Z Velet L Remer EM . Decline in renal function after partial nephrectomy: etiology and prevention. J Urol. (2015) 193:1889–98. doi: 10.1016/j.juro.2015.01.093 25637858

[10] Smith ZL Malkowicz SB . Tumor enucleation for renal cell carcinoma. J Kidney Cancer VHL. (2015) 2:64–9. doi: 10.15586/jkcvhl.2015.27 28326260 PMC5345541

[11] Laryngakis NA Guzzo TJ . Tumor enucleation for small renal masses. Curr Opin Urol. (2012) 22:365–71. doi: 10.1097/MOU.0b013e3283551f84 22647650

[12] Laryngakis NA Van Arsdalen KN Guzzo TJ Malkowicz SB . Tumor enucleation: a safe treatment alternative for renal cell carcinoma. Expert Rev Anticancer Ther. (2011) 11:893–9. doi: 10.1586/era.11.68 21707286

[13] Di Lascio G Sciarra A Del Giudice F Salciccia S Busetto GM Berardinis ED . Which factors can influence post-operative renal function preservation after nephron-sparing surgery for kidney cancer: a critical review. Cent Eur J Urol. (2022) 75:14–27. doi: 10.5173/ceju.2021.0256 35591956 PMC9074067

[14] Tsai S-H Tseng P-T Sherer BA Lai Y-C Lin P-Y Wu C-K . Open versus robotic partial nephrectomy: systematic review and meta-analysis of contemporary studies. Int J Med Robot. (2019) 15:e1963. doi: 10.1002/rcs.1963 30265760

[15] Shuch B Singer EA Bratslavsky G . The surgical approach to multifocal renal cancers: hereditary syndromes, ipsilateral multifocality, and bilateral tumors. Urol Clin North Am. (2012) 39:133–48. doi: 10.1016/j.ucl.2012.01.006 22487757

[16] Xu C Lin C Xu Z Feng S Zheng Y . Tumor enucleation vs. partial nephrectomy for T1 renal cell carcinoma: a systematic review and meta-analysis. Front Oncol. (2019) 9:473. doi: 10.3389/fonc.2019.00473 31214511 PMC6557988

[17] Minervini A Campi R Sessa F Derweesh I Kaouk JH Mari A . Positive surgical margins and local recurrence after simple enucleation and standard partial nephrectomy for Malignant renal tumors: systematic review of the literature and meta-analysis of prevalence. Minerva Urol Nefrol. (2017) 69:523–38. doi: 10.23736/S0393-2249.17.02864-8 28124871

[18] Chung HC Kang TW Lee JY Hwang EC Park HJ Hwang JE . Tumor enucleation for the treatment of T1 renal tumors: a systematic review and meta-analysis. Investig Clin Urol. (2022) 63:126–39. doi: 10.4111/icu.20210361 35244986 PMC8902429

[19] Page MJ McKenzie JE Bossuyt PM Boutron I Hoffmann TC Mulrow CD . The PRISMA 2020 statement: an updated guideline for reporting systematic reviews. BMJ. (2021) 372:n71. doi: 10.1136/bmj.n71 33782057 PMC8005924

[20] Dindo D Demartines N Clavien P-A . Classification of surgical complications: a new proposal with evaluation in a cohort of 6336 patients and results of a survey. Ann Surg. (2004) 240:205–13. doi: 10.1097/01.sla.0000133083.54934.ae 15273542 PMC1360123

[21] Sterne JAC Savović J Page MJ Elbers RG Blencowe NS Boutron I . RoB 2: a revised tool for assessing risk of bias in randomised trials. BMJ. (2019) 366:l4898. doi: 10.1136/bmj.l4898 31462531

[22] Luo D Wan X Liu J Tong T . Optimally estimating the sample mean from the sample size, median, mid-range, and/or mid-quartile range. Stat Methods Med Res. (2018) 27:1785–805. doi: 10.1177/0962280216669183 27683581

[23] Wan X Wang W Liu J Tong T . Estimating the sample mean and standard deviation from the sample size, median, range and/or interquartile range. BMC Med Res Methodol. (2014) 14:135. doi: 10.1186/1471-2288-14-135 25524443 PMC4383202

[24] Huang J Zhang J Wang Y Kong W Xue W Liu D . Comparing zero ischemia laparoscopic radio frequency ablation assisted tumor enucleation and laparoscopic partial nephrectomy for clinical T1a renal tumor: a randomized clinical trial. J Urol. (2016) 195:1677–83. doi: 10.1016/j.juro.2015.12.115 26905020

[25] Lu Q Zhao X Zhang S Wang G Ji C Liu G . Robot-assisted simple enucleation versus standard robot-assisted partial nephrectomy for low- or intermediate-complexity, clinical T1 renal tumors: a randomized controlled noninferiority trial. Eur Urol Oncol. (2024) 7:275–81. doi: 10.1016/j.euo.2023.07.019 37598032

[26] Wu X Chen W Huang J Zhang J Liu D Huang Y . Zero ischemia laparoscopic microwave ablation assisted enucleation vs. laparoscopic partial nephrectomy in clinical T1a renal tumor: a randomized clinical trial. Transl Cancer Res. (2020) 9:194–202. doi: 10.21037/tcr.2019.12.73 35117173 PMC8798795

[27] Longo N Minervini A Antonelli A Bianchi G Bocciardi AM Cunico SC . Simple enucleation versus standard partial nephrectomy for clinical T1 renal masses: perioperative outcomes based on a matched-pair comparison of 396 patients (RECORd project). Eur J Surg Oncol. (2014) 40:762–8. doi: 10.1016/j.ejso.2014.01.007 24529794

[28] Minoda R Takagi T Yoshida K Kondo T Tanabe K . Comparison of surgical outcomes between enucleation and standard resection in robot-assisted partial nephrectomy for completely endophytic renal tumors through a 1:1 propensity score-matched analysis. J Endourol. (2021) 35:1779–84. doi: 10.1089/end.2021.0213 34235961

[29] Takagi T Kondo T Tachibana H Iizuka J Omae K Yoshida K . Comparison of surgical outcomes between resection and enucleation in robot-assisted laparoscopic partial nephrectomy for renal tumors according to the surface-intermediate-base margin score: a propensity score-matched study. J Endourol. (2017) 31:756–61. doi: 10.1089/end.2017.0260 28537438

[30] Culpan M Atis G Sanli O Bozkurt Y Atmaca AF Semerci B . Comparison of tumor enucleation and standard partial nephrectomy according to trifecta outcomes: a multicenter study by the Turkish Academy of Urology, Uro-Oncology Working Group. J Invest Surg. (2022) 35:1112–8. doi: 10.1080/08941939.2021.2015490 34913804

[31] Dobrota F Andras I Gherle B Vesa SC Stanca DV Coman I . 3D laparoscopic enucleation vs standard partial nephrectomy for cT1 renal masses: assessment of functional outcomes at 1-year follow-up. Ann Ital Chir. (2020) 91:321–6. Available online at: https://pubmed.ncbi.nlm.nih.gov/32879058/. 32879058

[32] Blackwell RH Li B Kozel Z Zhang Z Zhao J Dong W . Functional implications of renal tumor enucleation relative to standard partial nephrectomy. Urology. (2017) 99:162–8. doi: 10.1016/j.urology.2016.07.048 27614120

[33] Deng YM Zhao XZ Ye CX Zhang Q Li XG Zhang GT . Laparoscopic tumor enucleation for renal cell carcinoma of T1aN0M0 stage: the experience in a single centre. J Minimally Invasive Urol. (2015) 4:11–5. [in Chinese] Available online at: https://kns.cnki.net/kcms2/article/abstract?v=nTS8fe6ew4omPLhkI8dg56DycY_d4XQ6YkKrNouCQSJ6PnJLb1Ef9MwlbtfOChXVcwlvH9IMvINOENs8Ge_i3lJ-Z2kmSkkIdMS1ednugsk9LEYzRe7JC2JyS1aC6sVcbEg5h0dBJPxwSVFnk7VLUGU2qZAF5fGC0rt78O_ZqkLzFn29jXFayLg_9yCt7P9tChKba6d1wsY&uniplatform=NZKPT.

[34] Dong W Gupta GN Blackwell RH Wu J Suk-Ouichai C Shah A . Functional comparison of renal tumor enucleation versus standard partial nephrectomy. Eur Urol Focus. (2017) 3:437–43. doi: 10.1016/j.euf.2017.06.002 28753814

[35] Ellis JL Sontag-Milobsky I Chen VS Rac G Hartman NC Gorbonos A . Quantifying preserved renal volume and function in patients undergoing standard partial nephrectomy vs. tumor enucleation for localized renal tumors. Urol Oncol. (2024) 42:454.e1–7. doi: 10.1016/j.urolonc.2024.09.018 39370308

[36] Lei K Wang X Yang Z Zhong Y Liu Y Sun T . Robotic-assisted tumor enucleation versus robotic-assisted partial nephrectomy for intermediate and high complexity renal cell carcinoma: a single-institution experience. World J Surg Oncol. (2023) 21:175. doi: 10.1186/s12957-023-03060-3 37287019 PMC10249187

[37] Lu Q Zhao X Ji C Guo S Liu G Zhang S . Modified laparoscopic simple enucleation with single-layer suture technique versus standard laparoscopic partial nephrectomy for treating localized renal cell carcinoma. Int Urol Nephrol. (2017) 49:239–45. doi: 10.1007/s11255-016-1470-1 27896577

[38] Lu Z Zhou J Yang C Zhang L Tai S Yin Y . The feasibility and safety of modified robot-assisted enucleation for highly complex renal tumors: research on a surgical technique. Transl Cancer Res. (2019) 8:761–9. doi: 10.21037/tcr.2019.04.20 35116814 PMC8798219

[39] Mukkamala A Allam CL Ellison JS Hafez KS Miller DC Montgomery JS . Tumor enucleation vs sharp excision in minimally invasive partial nephrectomy: technical benefit without impact on functional or oncologic outcomes. Urology. (2014) 83:1294–9. doi: 10.1016/j.urology.2014.02.007 24713137

[40] Zhao X Lu Q Ji C Liu G Qiu X Zhang S . Trifecta outcomes of modified robot-assisted simple enucleation and standard robot-assisted partial nephrectomy for treating clinical T1b renal cell carcinoma. Transl Androl Urol. (2021) 10:1080–7. doi: 10.21037/tau-20-1153 33850743 PMC8039591

[41] Minervini A Di Cristofano C Lapini A Marchi M Lanzi F Giubilei G . Histopathologic analysis of peritumoral pseudocapsule and surgical margin status after tumor enucleation for renal cell carcinoma. Eur Urol. (2009) 55:1410–8. doi: 10.1016/j.eururo.2008.07.038 18692300

[42] Bertolo R Pecoraro A Carbonara U Amparore D Diana P Muselaers S . Resection techniques during robotic partial nephrectomy: a systematic review. Eur Urol Open Sci. (2023) 52:7–21. doi: 10.1016/j.euros.2023.03.008 37182118 PMC10172691

[43] García AG León TG . Simple enucleation for renal tumors: indications, techniques, and results. Curr Urol Rep. (2016) 17:7. doi: 10.1007/s11934-015-0560-4 26728315

[44] Swavely NR Anele UA Porpiglia F Mir MC Hampton LJ Autorino R . Optimization of renal function preservation during robotic partial nephrectomy. Ther Adv Urol. (2019) 11:1756287218815819. doi: 10.1177/1756287218815819 30671138 PMC6329014

[45] Marconi L Desai MM Ficarra V Porpiglia F Van Poppel H . Renal preservation and partial nephrectomy: patient and surgical factors. Eur Urol Focus. (2016) 2:589–600. doi: 10.1016/j.euf.2017.02.012 28723490

[46] Aguilar Palacios D Wilson B Ascha M Campbell RA Song S DeWitt-Foy ME . New baseline renal function after radical or partial nephrectomy: a simple and accurate predictive model. J Urol. (2021) 205:1310–20. doi: 10.1097/JU.0000000000001549 33356481

[47] Larcher A Muttin F Peyronnet B De Naeyer G Khene Z-E Dell’Oglio P . The learning curve for robot-assisted partial nephrectomy: impact of surgical experience on perioperative outcomes. Eur Urol. (2019) 75:253–6. doi: 10.1016/j.eururo.2018.08.042 30243798

[48] Knight SR . The value of systematic reviews and meta-analyses in surgery. Eur Surg Res. (2021) 62: 221–228. doi: 10.1159/000519593 34710877

[49] Kalkum E Klotz R Seide S Hüttner FJ Kowalewski K-F Nickel F . Systematic reviews in surgery—recommendations from the Study Center of the German Society of Surgery. Langenbecks Arch Surg. (2021) 406:1723–31. doi: 10.1007/s00423-021-02204-x 34129108 PMC8481197

[50] Henderickx MMEL Baldew SV Marconi L Van Dijk MD Van Etten-Jamaludin FS Lagerveld BW . Surgical margins after partial nephrectomy as prognostic factor for the risk of local recurrence in pT1 RCC: a systematic review and narrative synthesis. World J Urol. (2022) 40:2169–79. doi: 10.1007/s00345-022-04016-0 35503118 PMC9427912

[51] Bex A Abu-Ghanem Y Albiges L Bonn S Campi R Capitanio U . EAU guidelines on renal cell carcinoma. In: Eau Guidelines. EAU Guidelines Office, Arnhem, The Netherlands (2026), ISBN: 978-94-92671-32-5.

[52] Bai R Gao L Wang J Jiang Q . Positive surgical margins may not affect the survival of patients with renal cell carcinoma after partial nephrectomy: a meta-analysis based on 39 studies. Front Oncol. (2022) 12:945166. doi: 10.3389/fonc.2022.945166 36033492 PMC9399599

[53] Guliev B Komyakov B Shevnin M Agagyulov M Talyshinskii A . Management of persistent urine leak after partial nephrectomy: a case series. Curr Urol. (2024) 18:155–8. doi: 10.1097/CU9.0000000000000136 39176290 PMC11338002

[54] Peyton CC Hajiran A Morgan K Azizi M Tang D Chipollini J . Urinary leak following partial nephrectomy: a contemporary review of 975 cases. Can J Urol. (2020) 27:10118–24. Available online at: https://pubmed.ncbi.nlm.nih.gov/32065869/. 32065869

[55] Campbell SC Novick AC Streem SB Klein E Licht M . Complications of nephron sparing surgery for renal tumors. J Urol. (1994) 151:1177–80. doi: 10.1016/S0022-5347(17)35207-2 8158754

