# Supplementary material for: Tumor enucleation versus conventional partial nephrectomy for localized renal tumors: a systematic review and meta-analysis of functional, perioperative, and margin outcomes
Source: Front Oncol. 2026 Jun 26;16:1853974. doi: 10.3389/fonc.2026.1853974 (PMC13349772; doi:10.3389/fonc.2026.1853974)
Supplement: Supplementary Figure 1 — Leave-one-out sensitivity analyses of pooled outcomes. (A) Postoperative eGFR. (B) Absolute ΔeGFR. (C) Positive surgical margin. (D) Major complications. (E) Warm ischemia time. (F) Operative time. (G) Estimated blood loss. [file DataSheet1.pdf]

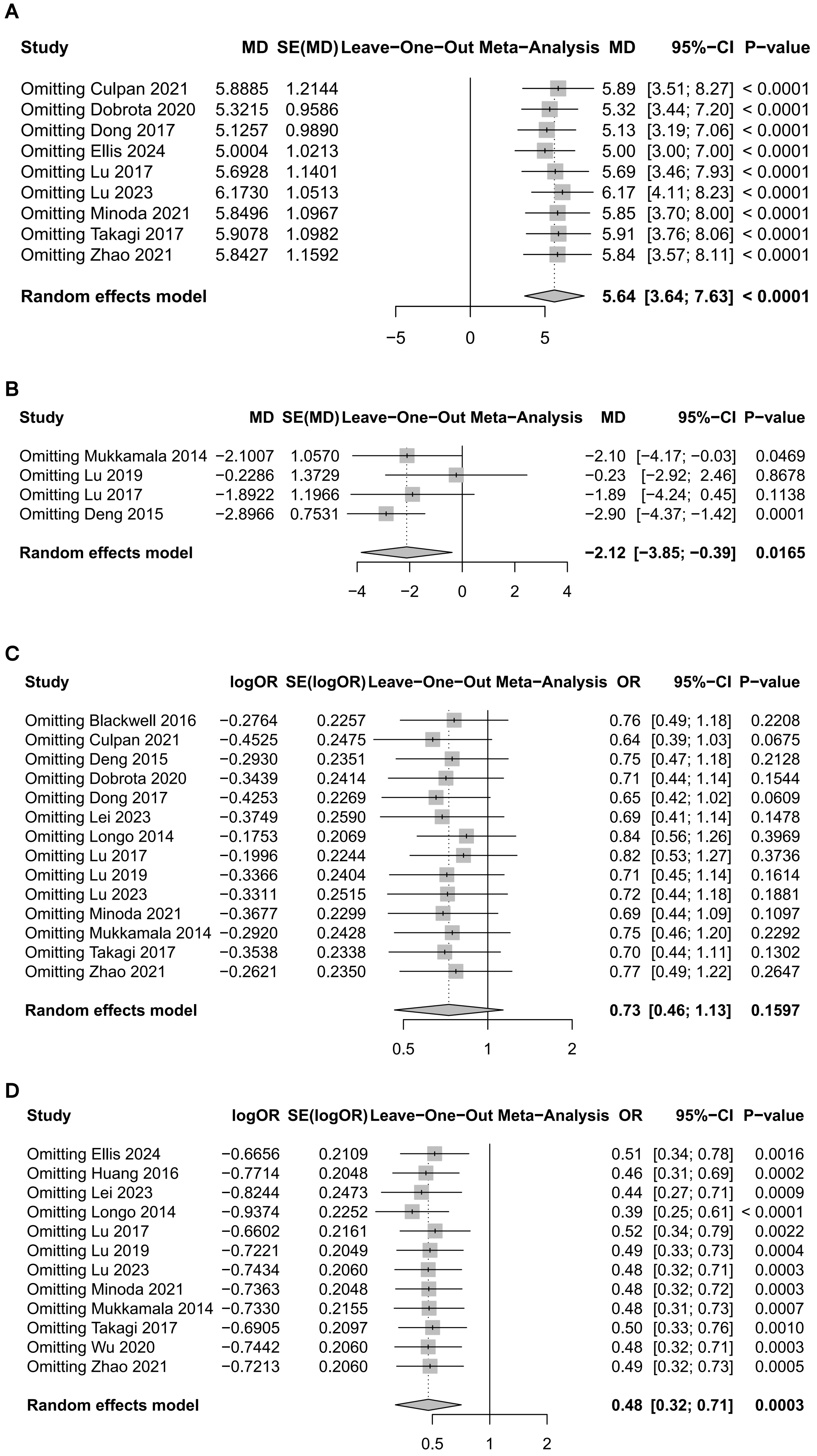
A
Study MD SE(MD) Leave−One−Out Meta−Analysis MD 95%−CI P−value
Omitting Culpan 2021 5.8885 1.2144 5.89 [3.51; 8.27] < 0.0001
Omitting Dobrota 2020 5.3215 0.9586 5.32 [3.44; 7.20] < 0.0001
Omitting Dong 2017 5.1257 0.9890 5.13 [3.19; 7.06] < 0.0001
Omitting Ellis 2024 5.0004 1.0213 5.00 [3.00; 7.00] < 0.0001
Omitting Lu 2017 5.6928 1.1401 5.69 [3.46; 7.93] < 0.0001
Omitting Lu 2023 6.1730 1.0513 6.17 [4.11; 8.23] < 0.0001
Omitting Minoda 2021 5.8496 1.0967 5.85 [3.70; 8.00] < 0.0001
Omitting Takagi 2017 5.9078 1.0982 5.91 [3.76; 8.06] < 0.0001
Omitting Zhao 2021 5.8427 1.1592 5.84 [3.57; 8.11] < 0.0001
Random effects model 5.64 [3.64; 7.63] < 0.0001
−5
0
5
B
Study MD SE(MD) Leave−One−Out Meta−Analysis MD 95%−CI P−value
Omitting Mukkamala 2014 −2.1007 1.0570 −2.10 [−4.17; −0.03] 0.0469
Omitting Lu 2019 −0.2286 1.3729 −0.23 [−2.92; 2.46] 0.8678
Omitting Lu 2017 −1.8922 1.1966 −1.89 [−4.24; 0.45] 0.1138
Omitting Deng 2015 −2.8966 0.7531 −2.90 [−4.37; −1.42] 0.0001
Random effects model −2.12 [−3.85; −0.39] 0.0165
−4
−2
0
2
4
C
Study logOR SE(logOR) Leave−One−Out Meta−Analysis OR 95%−CI P−value
Omitting Blackwell 2016 −0.2764 0.2257 0.76 [0.49; 1.18] 0.2208
Omitting Culpan 2021 −0.4525 0.2475 0.64 [0.39; 1.03] 0.0675
Omitting Deng 2015 −0.2930 0.2351 0.75 [0.47; 1.18] 0.2128
Omitting Dobrota 2020 −0.3439 0.2414 0.71 [0.44; 1.14] 0.1544
Omitting Dong 2017 −0.4253 0.2269 0.65 [0.42; 1.02] 0.0609
Omitting Lei 2023 −0.3749 0.2590 0.69 [0.41; 1.14] 0.1478
Omitting Longo 2014 −0.1753 0.2069 0.84 [0.56; 1.26] 0.3969
Omitting Lu 2017 −0.1996 0.2244 0.82 [0.53; 1.27] 0.3736
Omitting Lu 2019 −0.3366 0.2404 0.71 [0.45; 1.14] 0.1614
Omitting Lu 2023 −0.3311 0.2515 0.72 [0.44; 1.18] 0.1881
Omitting Minoda 2021 −0.3677 0.2299 0.69 [0.44; 1.09] 0.1097
Omitting Mukkamala 2014 −0.2920 0.2428 0.75 [0.46; 1.20] 0.2292
Omitting Takagi 2017 −0.3538 0.2338 0.70 [0.44; 1.11] 0.1302
Omitting Zhao 2021 −0.2621 0.2350 0.77 [0.49; 1.22] 0.2647
Random effects model 0.73 [0.46; 1.13] 0.1597
0.5
1
2
D
Study logOR SE(logOR) Leave−One−Out Meta−Analysis OR 95%−CI P−value
Omitting Ellis 2024 −0.6656 0.2109 0.51 [0.34; 0.78] 0.0016
Omitting Huang 2016 −0.7714 0.2048 0.46 [0.31; 0.69] 0.0002
Omitting Lei 2023 −0.8244 0.2473 0.44 [0.27; 0.71] 0.0009
Omitting Longo 2014 −0.9374 0.2252 0.39 [0.25; 0.61] < 0.0001
Omitting Lu 2017 −0.6602 0.2161 0.52 [0.34; 0.79] 0.0022
Omitting Lu 2019 −0.7221 0.2049 0.49 [0.33; 0.73] 0.0004
Omitting Lu 2023 −0.7434 0.2060 0.48 [0.32; 0.71] 0.0003
Omitting Minoda 2021 −0.7363 0.2048 0.48 [0.32; 0.72] 0.0003
Omitting Mukkamala 2014 −0.7330 0.2155 0.48 [0.31; 0.73] 0.0007
Omitting Takagi 2017 −0.6905 0.2097 0.50 [0.33; 0.76] 0.0010
Omitting Wu 2020 −0.7442 0.2060 0.48 [0.32; 0.71] 0.0003
Omitting Zhao 2021 −0.7213 0.2060 0.49 [0.32; 0.73] 0.0005
Random effects model 0.48 [0.32; 0.71] 0.0003
0.5
1
2

E

Study	MD	SE(MD)	MD	95%-CI	P-value
Omitting Blackwell 2016	−2.0605	0.9507	−2.06	[−3.92; −0.20]	0.0302
Omitting Deng 2015	−2.2414	0.9294	−2.24	[−4.06; −0.42]	0.0159
Omitting Dong 2017	−2.2791	0.9314	−2.28	[−4.10; −0.45]	0.0144
Omitting Ellis 2024	−1.1839	0.7306	−1.18	[−2.62; 0.25]	0.1051
Omitting Lei 2023	−1.7445	0.9573	−1.74	[−3.62; 0.13]	0.0684
Omitting Longo 2014	−2.1923	0.9544	−2.19	[−4.06; −0.32]	0.0216
Omitting Lu 2017	−1.9673	0.9804	−1.97	[−3.89; −0.05]	0.0448
Omitting Lu 2019	−1.8865	1.0087	−1.89	[−3.86; 0.09]	0.0615
Omitting Lu 2023	−2.0723	0.9926	−2.07	[−4.02; −0.13]	0.0368
Omitting Minoda 2021	−2.2243	0.9244	−2.22	[−4.04; −0.41]	0.0161
Omitting Mukkamala 2014	−1.8299	0.9503	−1.83	[−3.69; 0.03]	0.0542
Omitting Takagi 2017	−2.3836	0.9154	−2.38	[−4.18; −0.59]	0.0092
Omitting Zhao 2021	−1.9145	0.9600	−1.91	[−3.80; −0.03]	0.0461
**Random effects model**			**−2.00**	**[−3.76; −0.23]**	**0.0265**

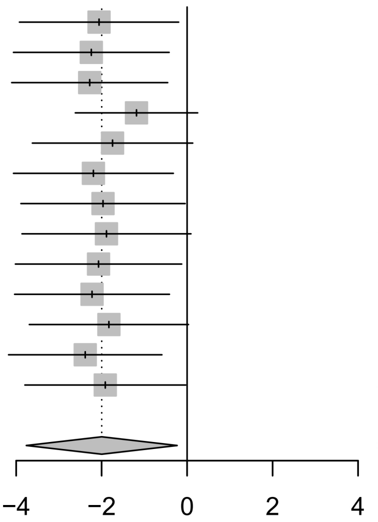

F

Study	MD	SE(MD)	MD	95%-CI	P-value
Omitting Culpan 2021	−15.3794	2.2642	−15.38	[−19.82; −10.94]	< 0.0001
Omitting Deng 2015	−13.6477	3.4792	−13.65	[−20.47; −6.83]	< 0.0001
Omitting Huang 2016	−12.1410	3.3483	−12.14	[−18.70; −5.58]	0.0003
Omitting Lei 2023	−13.5156	3.8316	−13.52	[−21.03; −6.01]	0.0004
Omitting Longo 2014	−11.6039	3.3079	−11.60	[−18.09; −5.12]	0.0005
Omitting Lu 2017	−13.0997	3.4534	−13.10	[−19.87; −6.33]	0.0001
Omitting Lu 2019	−12.9689	4.0148	−12.97	[−20.84; −5.10]	0.0012
Omitting Lu 2023	−13.1068	3.5608	−13.11	[−20.09; −6.13]	0.0002
Omitting Minoda 2021	−12.0177	3.3314	−12.02	[−18.55; −5.49]	0.0003
Omitting Mukkamala 2014	−11.3968	3.2542	−11.40	[−17.77; −5.02]	0.0005
Omitting Takagi 2017	−13.4740	3.3784	−13.47	[−20.10; −6.85]	< 0.0001
Omitting Wu 2020	−12.4747	3.4545	−12.47	[−19.25; −5.70]	0.0003
Omitting Zhao 2021	−12.5967	3.3771	−12.60	[−19.22; −5.98]	0.0002
**Random effects model**			**−12.89**	**[−19.24; −6.54]**	**< 0.0001**

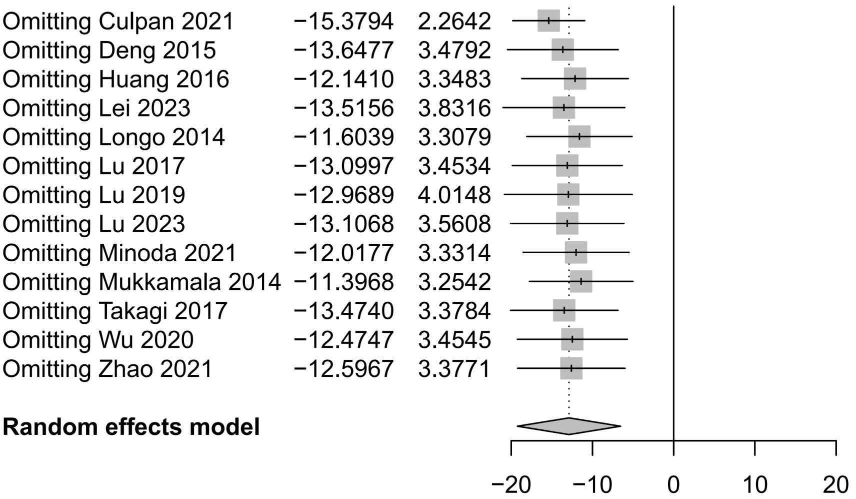

G

Study	MD	SE(MD)	MD	95%-CI	P-value
Omitting Culpan 2021	−51.4396	14.8702	−51.44	[−80.58; −22.29]	0.0005
Omitting Deng 2015	−45.9799	14.3338	−45.98	[−74.07; −17.89]	0.0013
Omitting Dong 2017	−37.9812	9.8109	−37.98	[−57.21; −18.75]	0.0001
Omitting Ellis 2024	−41.2251	13.7695	−41.23	[−68.21; −14.24]	0.0028
Omitting Huang 2016	−43.7406	14.1126	−43.74	[−71.40; −16.08]	0.0019
Omitting Lei 2023	−48.4979	17.0143	−48.50	[−81.85; −15.15]	0.0044
Omitting Longo 2014	−47.5307	14.8096	−47.53	[−76.56; −18.50]	0.0013
Omitting Lu 2017	−51.3017	14.1165	−51.30	[−78.97; −23.63]	0.0003
Omitting Lu 2019	−46.8382	14.6729	−46.84	[−75.60; −18.08]	0.0014
Omitting Lu 2023	−53.0809	14.0421	−53.08	[−80.60; −25.56]	0.0002
Omitting Minoda 2021	−48.6124	14.4026	−48.61	[−76.84; −20.38]	0.0007
Omitting Mukkamala 2014	−48.2716	13.8955	−48.27	[−75.51; −21.04]	0.0005
Omitting Takagi 2017	−49.3500	13.9358	−49.35	[−76.66; −22.04]	0.0004
Omitting Wu 2020	−46.6469	14.6029	−46.65	[−75.27; −18.03]	0.0014
Omitting Zhao 2021	−47.7614	14.1260	−47.76	[−75.45; −20.07]	0.0007
**Random effects model**			**−47.34**	**[−74.14; −20.55]**	**0.0005**

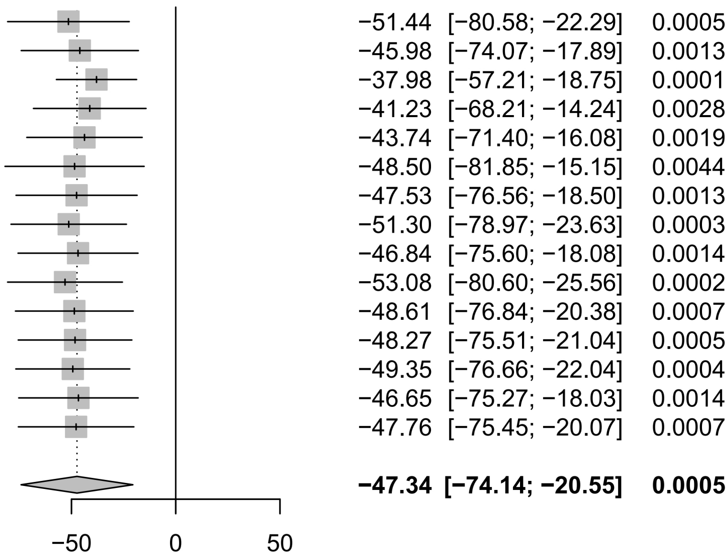

Figure S1. Leave-one-out sensitivity analyses of pooled outcomes.
(A) Postoperative eGFR (B) Absolute ΔeGFR (C) Positive surgical margin (D) Major complications (E) Warm ischemia time (F) Operative time (G) Estimated blood loss
