# Supplementary material for: Tumor enucleation versus conventional partial nephrectomy for localized renal tumors: a systematic review and meta-analysis of functional, perioperative, and margin outcomes
Source: Front Oncol. 2026 Jun 26;16:1853974. doi: 10.3389/fonc.2026.1853974 (PMC13349772; doi:10.3389/fonc.2026.1853974)
Supplement: Supplementary Figure 2 — Subgroup analyses according to study design. (A) Postoperative eGFR. (B) Positive surgical margin. (C) Major complications. (D) Warm ischemia time. (E) Operative time. (F) Estimated blood loss. [file DataSheet2.pdf]

A

Study or Subgroup	TE Mean	TE SD	TE Total	PN Mean	PN SD	PN Total	Weight	Mean Difference IV, Random, 95% CI
**3.1.1 RCT**								
Lu 2023	99.5	21.7	190	97.9	23.5	190	14.4%	1.60 [−2.95, 6.15]
**Subtotal (95% CI)**			**190**			**190**	**14.4%**	**1.60 [−2.95, 6.15]**
Heterogeneity: Not applicable								
Test for overall effect: Z = 0.69 (P = 0.49)								
**3.1.2 Other**								
Culpan 2021	81.4	30	848	76.7	26.1	222	16.2%	4.70 [0.72, 8.68]
Dobrota 2020	82.4	20.4	29	69.9	21.7	54	5.7%	12.50 [3.09, 21.91]
Dong 2017	79	23.7	71	68	21.5	373	10.9%	11.00 [5.07, 16.93]
Ellis 2024	78.2	24.1	168	69.3	24.2	252	14.0%	8.90 [4.19, 13.61]
Lu 2017	109.6	36.3	280	104.2	28.2	105	9.0%	5.40 [−1.47, 12.27]
Minoda 2021	62	17	45	63	20	45	7.7%	−1.00 [−8.67, 6.67]
Takagi 2017	65	14	45	62	17	45	9.8%	3.00 [−3.43, 9.43]
Zhao 2021	87.7	18.4	139	83.3	17.8	64	12.3%	4.40 [−0.93, 9.73]
**Subtotal (95% CI)**			**1625**			**1160**	**85.6%**	**6.02 [3.42, 8.63]**
Heterogeneity: $Tau^2 = 4.83$; $Chi^2 = 10.84$, df = 7 (P = 0.15); $I^2 = 35\%$								
Test for overall effect: Z = 4.54 (P < 0.00001)								
**Total (95% CI)**			**1815**			**1350**	**100.0%**	**5.38 [2.87, 7.90]**
Heterogeneity: $Tau^2 = 6.02$; $Chi^2 = 13.86$, df = 8 (P = 0.09); $I^2 = 42\%$								
Test for overall effect: Z = 4.19 (P < 0.0001)								
Test for subgroup differences: $Chi^2 = 2.74$, df = 1 (P = 0.10), $I^2 = 63.5\%$								

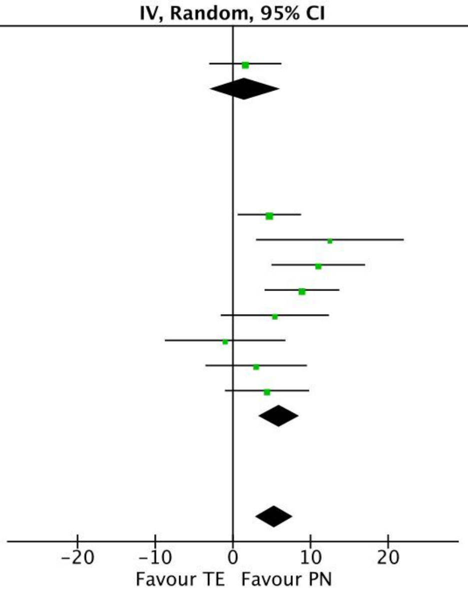

B

Study or Subgroup	TE Events	TE Total	PN Events	PN Total	Weight	Risk Ratio M-H, Random, 95% CI
**3.2.1 RCT**						
Lu 2023	4	190	6	190	8.6%	0.67 [0.19, 2.32]
**Subtotal (95% CI)**		**190**		**190**	**8.6%**	**0.67 [0.19, 2.32]**
Total events	4		6			
Heterogeneity: Not applicable						
Test for overall effect: Z = 0.64 (P = 0.52)						
**3.2.2 Other**						
Blackwell 2016	0	57	3	53	2.0%	0.13 [0.01, 2.52]
Culpan 2021	62	848	13	222	20.2%	1.25 [0.70, 2.23]
Deng 2015	1	94	3	85	3.3%	0.30 [0.03, 2.84]
Dobrota 2020	1	29	2	54	3.0%	0.93 [0.09, 9.84]
Dong 2017	6	71	18	373	13.5%	1.75 [0.72, 4.26]
Lei 2023	6	135	11	224	12.1%	0.91 [0.34, 2.39]
Longo 2014	2	147	10	145	6.5%	0.20 [0.04, 0.88]
Lu 2017	5	280	6	105	9.5%	0.31 [0.10, 1.00]
Lu 2019	1	94	1	72	2.3%	0.77 [0.05, 12.04]
Minoda 2021	3	45	1	45	3.3%	3.00 [0.32, 27.76]
Mukkamala 2014	2	86	26	516	7.1%	0.46 [0.11, 1.91]
Takagi 2017	1	45	0	45	1.7%	3.00 [0.13, 71.74]
Zhao 2021	3	139	4	64	6.8%	0.35 [0.08, 1.50]
**Subtotal (95% CI)**		**2070**		**2003**	**91.4%**	**0.73 [0.46, 1.17]**
Total events	93		98			
Heterogeneity: $Tau^2 = 0.20$; $Chi^2 = 17.21$, df = 12 (P = 0.14); $I^2 = 30\%$						
Test for overall effect: Z = 1.29 (P = 0.20)						
**Total (95% CI)**		**2260**		**2193**	**100.0%**	**0.74 [0.48, 1.14]**
Total events	97		104			
Heterogeneity: $Tau^2 = 0.15$; $Chi^2 = 17.33$, df = 13 (P = 0.18); $I^2 = 25\%$						
Test for overall effect: Z = 1.38 (P = 0.17)						
Test for subgroup differences: $Chi^2 = 0.02$, df = 1 (P = 0.89), $I^2 = 0\%$						

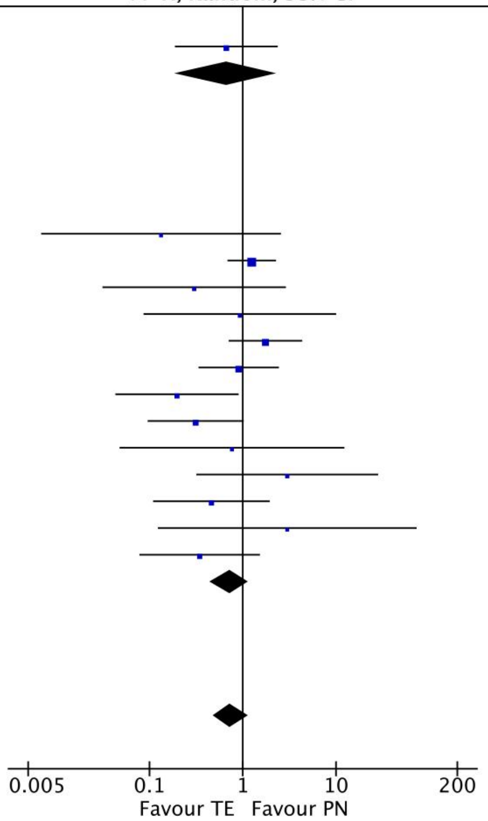

C

Study or Subgroup	TE Events	TE Total	PN Events	PN Total	Weight	Odds Ratio M-H, Random, 95% CI
**3.3.1 RCT**						
Huang 2016	1	44	0	45	1.5%	3.14 [0.12, 79.13]
Lu 2023	1	190	2	190	2.7%	0.50 [0.04, 5.53]
Wu 2020	1	90	2	93	2.7%	0.51 [0.05, 5.74]
**Subtotal (95% CI)**		**324**		**328**	**7.0%**	**0.75 [0.17, 3.40]**
Total events	3		4			
Heterogeneity: $Tau^2 = 0.00$; $Chi^2 = 0.97$, df = 2 (P = 0.62); $I^2 = 0\%$						
Test for overall effect: Z = 0.37 (P = 0.71)						
**3.3.2 Other**						
Ellis 2024	2	168	16	252	7.2%	0.18 [0.04, 0.78]
Lei 2023	12	135	33	224	32.5%	0.56 [0.28, 1.14]
Longo 2014	10	198	9	198	18.6%	1.12 [0.44, 2.81]
Lu 2017	5	280	7	105	11.6%	0.25 [0.08, 0.82]
Lu 2019	0	94	2	72	1.7%	0.15 [0.01, 3.16]
Minoda 2021	0	45	1	45	1.5%	0.33 [0.01, 8.22]
Mukkamala 2014	3	86	39	516	11.1%	0.44 [0.13, 1.46]
Takagi 2017	2	45	8	45	6.1%	0.22 [0.04, 1.08]
Zhao 2021	1	139	2	64	2.7%	0.22 [0.02, 2.52]
**Subtotal (95% CI)**		**1190**		**1521**	**93.0%**	**0.45 [0.30, 0.70]**
Total events	35		117			
Heterogeneity: $Tau^2 = 0.01$; $Chi^2 = 8.24$, df = 8 (P = 0.41); $I^2 = 3\%$						
Test for overall effect: Z = 3.63 (P = 0.0003)						
**Total (95% CI)**		**1514**		**1849**	**100.0%**	**0.48 [0.32, 0.71]**
Total events	38		121			
Heterogeneity: $Tau^2 = 0.00$; $Chi^2 = 9.57$, df = 11 (P = 0.57); $I^2 = 0\%$						
Test for overall effect: Z = 3.65 (P = 0.0003)						
Test for subgroup differences: $Chi^2 = 0.40$, df = 1 (P = 0.53), $I^2 = 0\%$						

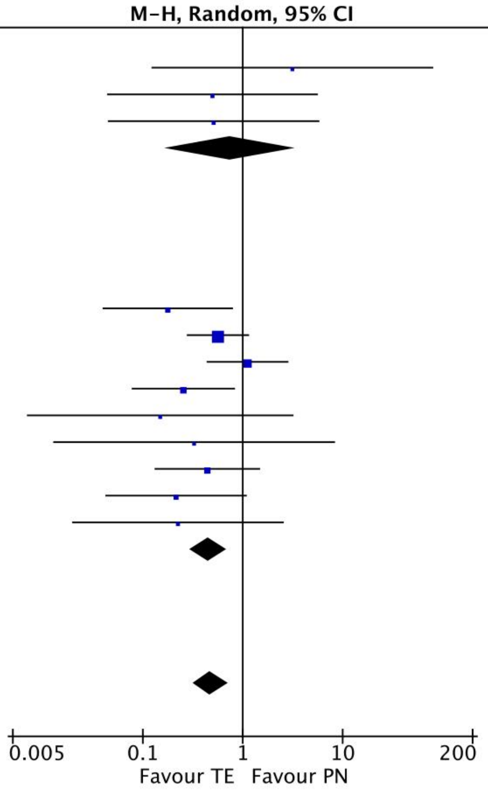

D

Study or Subgroup	TE Mean	TE SD	TE Total	PN Mean	PN SD	PN Total	Weight	Mean Difference IV, Random, 95% CI
**3.4.1 RCT**								
Lu 2023	20.67	5.93	190	21.67	5.19	190	8.4%	−1.00 [−2.12, 0.12]
**Subtotal (95% CI)**			**190**			**190**	**8.4%**	**−1.00 [−2.12, 0.12]**
Heterogeneity: Not applicable								
Test for overall effect: Z = 1.75 (P = 0.08)								
**3.4.2 Other**								
Blackwell 2016	24.33	6.67	57	25.5	5.93	53	7.5%	−1.17 [−3.53, 1.19]
Deng 2015	23.4	3.6	94	22.8	3.2	85	8.4%	0.60 [−0.40, 1.60]
Dong 2017	23	8.15	71	21.67	8.15	373	7.7%	1.33 [−0.74, 3.40]
Ellis 2024	10.7	10.1	168	23.4	11	252	7.8%	−12.70 [−14.74, −10.66]
Lei 2023	20.67	3.7	135	25.33	2.22	224	8.6%	−4.66 [−5.35, −3.97]
Longo 2014	18	5.5	198	17.8	6.9	198	8.3%	0.20 [−1.03, 1.43]
Lu 2017	23.2	6.2	280	25.4	6.6	105	8.2%	−2.20 [−3.66, −0.74]
Lu 2019	17.5	3.7	94	20.5	3.5	72	8.4%	−3.00 [−4.10, −1.90]
Minoda 2021	23	14	45	21	8	45	5.4%	2.00 [−2.71, 6.71]
Mukkamala 2014	23	10	86	27	11	516	7.5%	−4.00 [−6.32, −1.68]
Takagi 2017	24	12	45	20	6.4	45	6.0%	4.00 [0.03, 7.97]
Zhao 2021	21.2	6.4	139	24.1	6.9	64	7.8%	−2.90 [−4.90, −0.90]
**Subtotal (95% CI)**			**1412**			**2032**	**91.6%**	**−2.07 [−4.02, −0.13]**
Heterogeneity: $Tau^2 = 10.54$; $Chi^2 = 219.53$, df = 11 (P < 0.00001); $I^2 = 95\%$								
Test for overall effect: Z = 2.09 (P = 0.04)								
**Total (95% CI)**			**1602**			**2222**	**100.0%**	**−2.00 [−3.76, −0.23]**
Heterogeneity: $Tau^2 = 9.35$; $Chi^2 = 226.99$, df = 12 (P < 0.00001); $I^2 = 95\%$								
Test for overall effect: Z = 2.22 (P = 0.03)								
Test for subgroup differences: $Chi^2 = 0.88$, df = 1 (P = 0.35), $I^2 = 0\%$								

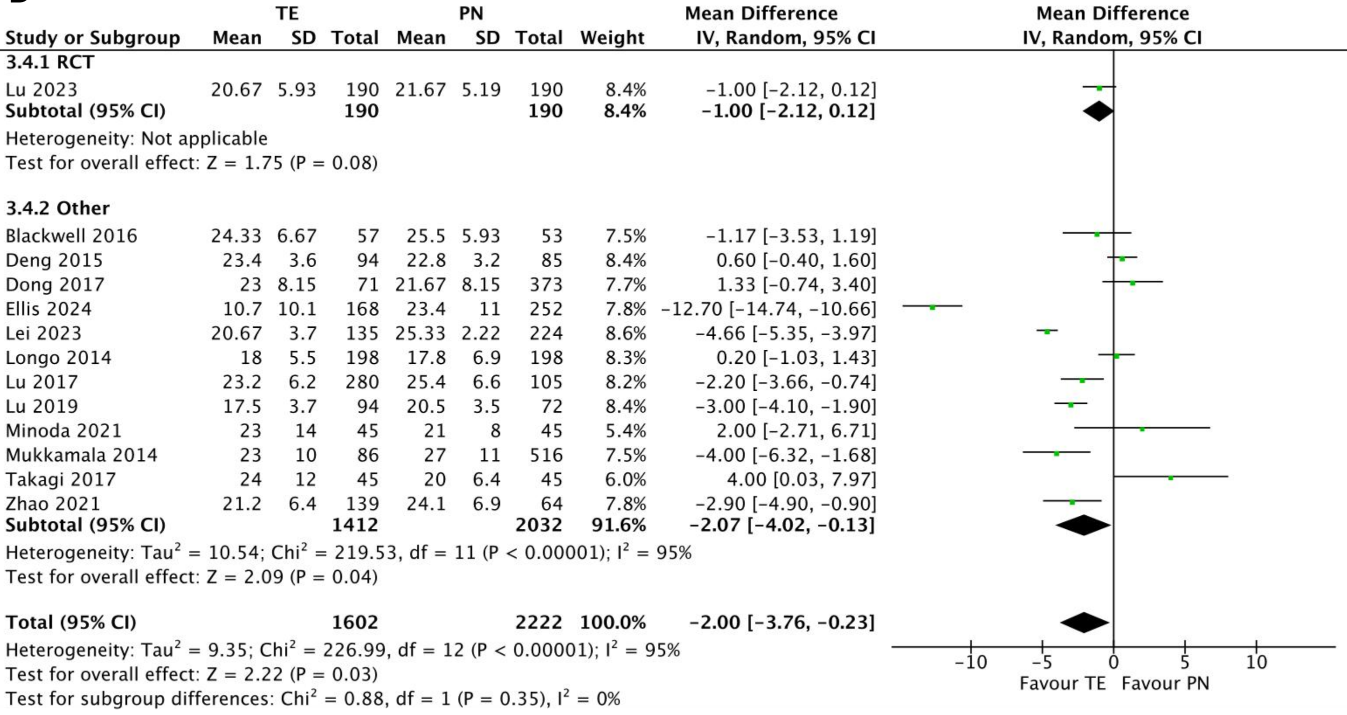

E

Study or Subgroup	TE Mean	TE SD	TE Total	PN Mean	PN SD	PN Total	Weight	Mean Difference IV, Random, 95% CI
**3.5.1 RCT**								
Huang 2016	100	38.8	44	124.8	42.8	45	5.9%	−24.80 [−41.76, −7.84]
Lu 2023	145	37	190	156.3	41.5	190	8.8%	−11.30 [−19.21, −3.39]
Wu 2020	101.8	36.2	90	119.8	36.2	93	8.0%	−18.00 [−28.49, −7.51]
**Subtotal (95% CI)**			**324**			**328**	**22.7%**	**−15.54 [−22.33, −8.75]**
Heterogeneity: $Tau^2 = 6.98$; $Chi^2 = 2.44$, df = 2 (P = 0.30); $I^2 = 18\%$								
Test for overall effect: Z = 4.48 (P < 0.00001)								
**3.5.2 Other**								
Culpan 2021	151.7	51.9	848	130	66.7	222	8.3%	21.70 [12.26, 31.14]
Deng 2015	146	30.1	94	151.1	30.1	85	8.5%	−5.10 [−13.93, 3.73]
Lei 2023	165	22.2	135	173.3	18.5	224	9.7%	−8.30 [−12.76, −3.84]
Longo 2014	121	44	198	147	42	198	8.6%	−26.00 [−34.47, −17.53]
Lu 2017	182.1	52.6	280	192.8	53	105	7.5%	−10.70 [−22.56, 1.16]
Lu 2019	107.1	10.3	94	121.3	11.5	72	9.9%	−14.20 [−17.58, −10.82]
Minoda 2021	140	44	45	167	40	45	5.8%	−27.00 [−44.37, −9.63]
Mukkamala 2014	159	58	86	191	58	516	7.1%	−32.00 [−45.24, −18.76]
Takagi 2017	189	37	45	193	43	45	6.0%	−4.00 [−20.57, 12.57]
Zhao 2021	197.7	54.6	139	215.6	61.6	64	5.7%	−17.90 [−35.51, −0.29]
**Subtotal (95% CI)**			**1964**			**1576**	**77.3%**	**−11.72 [−19.54, −3.89]**
Heterogeneity: $Tau^2 = 125.60$; $Chi^2 = 78.75$, df = 9 (P < 0.00001); $I^2 = 89\%$								
Test for overall effect: Z = 2.94 (P = 0.003)								
**Total (95% CI)**			**2288**			**1904**	**100.0%**	**−12.89 [−19.24, −6.54]**
Heterogeneity: $Tau^2 = 102.84$; $Chi^2 = 82.48$, df = 12 (P < 0.00001); $I^2 = 85\%$								
Test for overall effect: Z = 3.98 (P < 0.0001)								
Test for subgroup differences: $Chi^2 = 0.52$, df = 1 (P = 0.47), $I^2 = 0\%$								

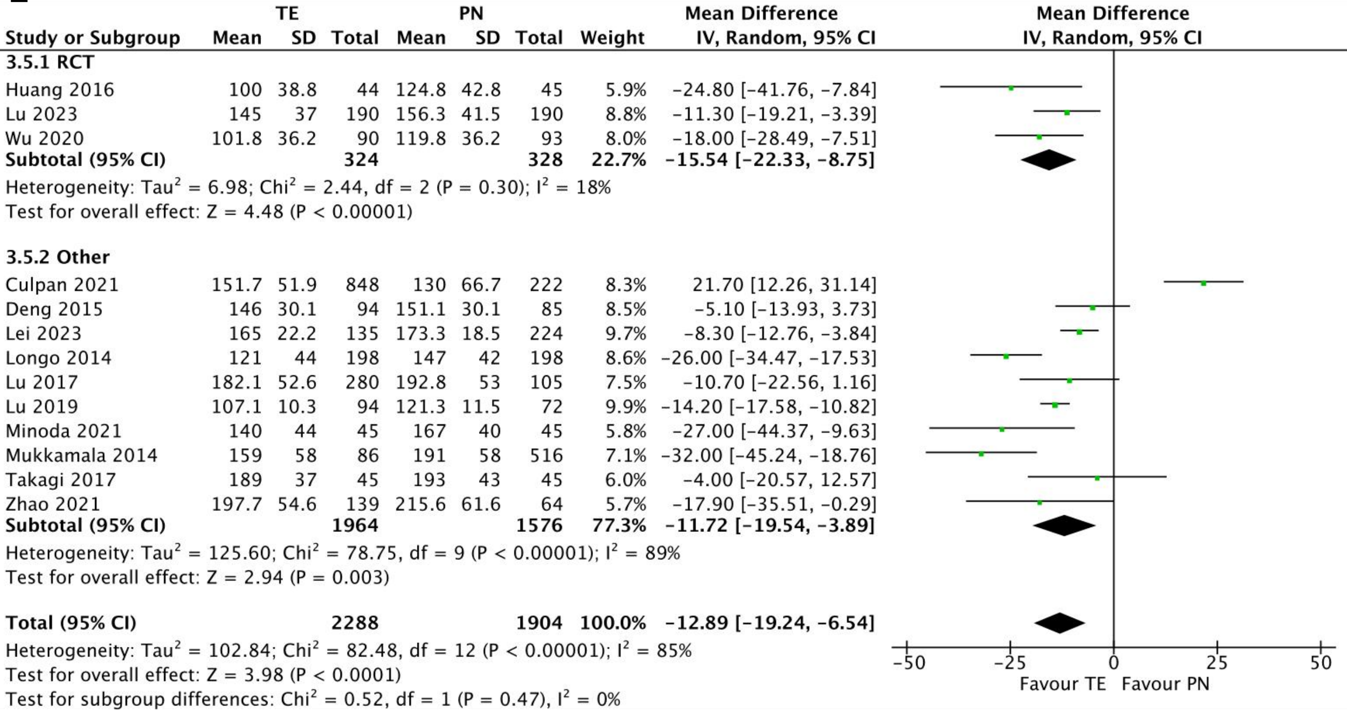

F

Study or Subgroup	TE Mean	TE SD	TE Total	PN Mean	PN SD	PN Total	Weight	Mean Difference IV, Random, 95% CI
**3.6.1 RCT**								
Huang 2016	122.5	70	44	220	145	45	6.7%	−97.50 [−144.64, −50.36]
Lu 2023	116.7	111.1	190	100	74.1	190	8.1%	16.70 [−2.29, 35.69]
Wu 2020	133.8	82.5	90	188.8	120	93	7.7%	−55.00 [−84.75, −25.25]
**Subtotal (95% CI)**			**324**			**328**	**22.5%**	**−42.74 [−109.34, 23.85]**
Heterogeneity: $Tau^2 = 3173.64$; $Chi^2 = 29.38$, df = 2 (P < 0.00001); $I^2 = 93\%$								
Test for overall effect: Z = 1.26 (P = 0.21)								
**3.6.2 Other**								
Culpan 2021	193.3	148.1	848	193.3	88.9	222	8.3%	0.00 [−15.37, 15.37]
Deng 2015	202.2	118	94	267.4	167.7	85	7.0%	−65.20 [−108.10, −22.30]
Dong 2017	56.7	59.3	71	200	148.1	373	8.1%	−143.30 [−163.70, −122.90]
Ellis 2024	97.5	216.5	168	235.8	333.1	252	6.4%	−138.30 [−190.87, −85.73]
Lei 2023	126.7	44.4	135	155	48.1	224	8.4%	−28.30 [−38.09, −18.51]
Longo 2014	177	128	198	221	131	198	7.9%	−44.00 [−69.51, −18.49]
Lu 2017	223.3	206.1	280	206.7	298.8	105	5.8%	16.60 [−45.44, 78.64]
Lu 2019	90.8	28.3	94	143.3	118.3	72	7.8%	−52.50 [−80.42, −24.58]
Minoda 2021	56	101	45	86	104	45	7.0%	−30.00 [−72.36, 12.36]
Mukkamala 2014	359	620	86	371	562	516	2.6%	−12.00 [−151.72, 127.72]
Takagi 2017	129	211	45	117	337	45	3.3%	12.00 [−104.17, 128.17]
Zhao 2021	230.5	207	139	269.8	273.3	64	5.1%	−39.30 [−114.58, 35.98]
**Subtotal (95% CI)**			**2203**			**2201**	**77.5%**	**−48.86 [−79.91, −17.80]**
Heterogeneity: $Tau^2 = 2312.13$; $Chi^2 = 149.98$, df = 11 (P < 0.00001); $I^2 = 93\%$								
Test for overall effect: Z = 3.08 (P = 0.002)								
**Total (95% CI)**			**2527**			**2529**	**100.0%**	**−47.34 [−74.14, −20.55]**
Heterogeneity: $Tau^2 = 2202.83$; $Chi^2 = 189.09$, df = 14 (P < 0.00001); $I^2 = 93\%$								
Test for overall effect: Z = 3.46 (P = 0.0005)								
Test for subgroup differences: $Chi^2 = 0.03$, df = 1 (P = 0.87), $I^2 = 0\%$								

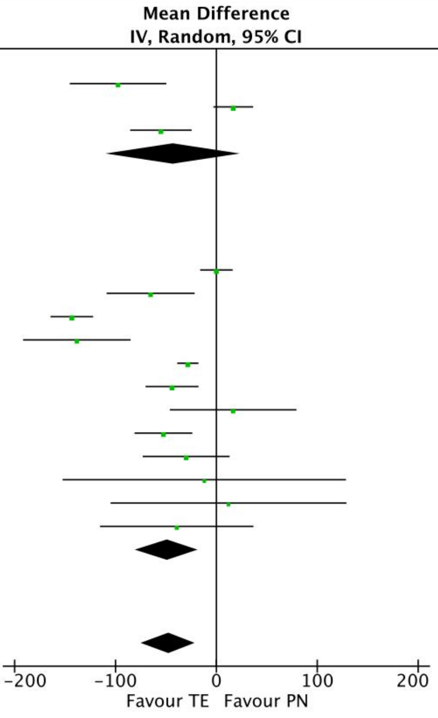

Figure S2. Subgroup analyses according to study design.
(A) Postoperative eGFR. (B) Positive surgical margin. (C) Major complications. (D) Warm ischemia time. (E) Operative time. (F) Estimated blood loss.
