# Supplementary material for: Tumor enucleation versus conventional partial nephrectomy for localized renal tumors: a systematic review and meta-analysis of functional, perioperative, and margin outcomes
Source: Front Oncol. 2026 Jun 26;16:1853974. doi: 10.3389/fonc.2026.1853974 (PMC13349772; doi:10.3389/fonc.2026.1853974)
Supplement: Supplementary Figure 3 — Subgroup analyses according to surgical platform. (A) Postoperative eGFR. (B) Absolute ΔeGFR. (C) Positive surgical margin. (D) Major complications. (E) Warm ischemia time. (F) Operative time. (G) Estimated blood loss. [file DataSheet3.pdf]

A

Study or Subgroup	TE Mean	TE SD	TE Total	PN Mean	PN SD	PN Total	Weight	Mean Difference IV, Random, 95% CI
**5.6.1 Robot Only**								
Blackwell 2016	73.3	17.3	57	68.9	20.7	53	7.6%	4.40 [−2.76, 11.56]
Ellis 2024	78.2	24.1	168	69.3	24.2	252	13.1%	8.90 [4.19, 13.61]
Lu 2023	99.5	21.7	190	97.9	23.5	190	13.6%	1.60 [−2.95, 6.15]
Minoda 2021	62	17	45	63	20	45	6.9%	−1.00 [−8.67, 6.67]
Takagi 2017	65	14	45	62	17	45	8.9%	3.00 [−3.43, 9.43]
Zhao 2021	87.7	18.4	139	83.3	17.8	64	11.4%	4.40 [−0.93, 9.73]
**Subtotal (95% CI)**			**644**			**649**	**61.5%**	**3.97 [1.20, 6.74]**
Heterogeneity: $Tau^2 = 3.36$; $Chi^2 = 6.98$, df = 5 (P = 0.22); $I^2 = 28\%$								
Test for overall effect: Z = 2.81 (P = 0.005)								
**5.6.2 Other**								
Culpan 2021	81.4	30	848	76.7	26.1	222	15.6%	4.70 [0.72, 8.68]
Dobrota 2020	82.4	20.4	29	69.9	21.7	54	4.9%	12.50 [3.09, 21.91]
Dong 2017	79	23.7	71	68	21.5	373	9.9%	11.00 [5.07, 16.93]
Lu 2017	109.6	36.3	280	104.2	28.2	105	8.1%	5.40 [−1.47, 12.27]
**Subtotal (95% CI)**			**1228**			**754**	**38.5%**	**7.49 [3.74, 11.24]**
Heterogeneity: $Tau^2 = 4.99$; $Chi^2 = 4.55$, df = 3 (P = 0.21); $I^2 = 34\%$								
Test for overall effect: Z = 3.92 (P < 0.0001)								
**Total (95% CI)**			**1872**			**1403**	**100.0%**	**5.30 [3.00, 7.59]**
Heterogeneity: $Tau^2 = 4.67$; $Chi^2 = 13.92$, df = 9 (P = 0.13); $I^2 = 35\%$								
Test for overall effect: Z = 4.52 (P < 0.00001)								
Test for subgroup differences: $Chi^2 = 2.20$, df = 1 (P = 0.14), $I^2 = 54.4\%$								

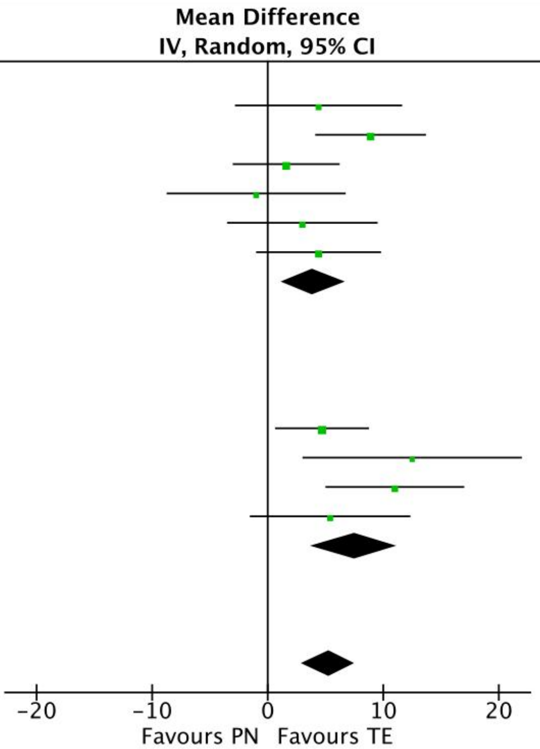

B

Study or Subgroup	TE Mean	TE SD	TE Total	PN Mean	PN SD	PN Total	Weight	Mean Difference IV, Random, 95% CI
**5.7.1 Robot Only**								
Lu 2019	9.49	4.09	94	12.65	5.67	72	64.3%	−3.16 [−4.71, −1.61]
**Subtotal (95% CI)**			**94**			**72**	**64.3%**	**−3.16 [−4.71, −1.61]**
Heterogeneity: Not applicable								
Test for overall effect: Z = 4.00 (P < 0.0001)								
**5.7.2 Other**								
Mukkamala 2014	2.8	25	86	2.3	60	516	5.3%	0.50 [−6.90, 7.90]
Lu 2017	2.6	33.6	280	3.5	26.83	105	6.8%	−0.90 [−7.37, 5.57]
Deng 2015	0.4	10.9	94	0.6	11.1	85	23.6%	−0.20 [−3.43, 3.03]
**Subtotal (95% CI)**			**460**			**706**	**35.7%**	**−0.23 [−2.92, 2.46]**
Heterogeneity: $Tau^2 = 0.00$; $Chi^2 = 0.08$, df = 2 (P = 0.96); $I^2 = 0\%$								
Test for overall effect: Z = 0.17 (P = 0.87)								
**Total (95% CI)**			**554**			**778**	**100.0%**	**−2.12 [−3.85, −0.39]**
Heterogeneity: $Tau^2 = 0.59$; $Chi^2 = 3.50$, df = 3 (P = 0.32); $I^2 = 14\%$								
Test for overall effect: Z = 2.40 (P = 0.02)								
Test for subgroup differences: $Chi^2 = 3.42$, df = 1 (P = 0.06), $I^2 = 70.8\%$								

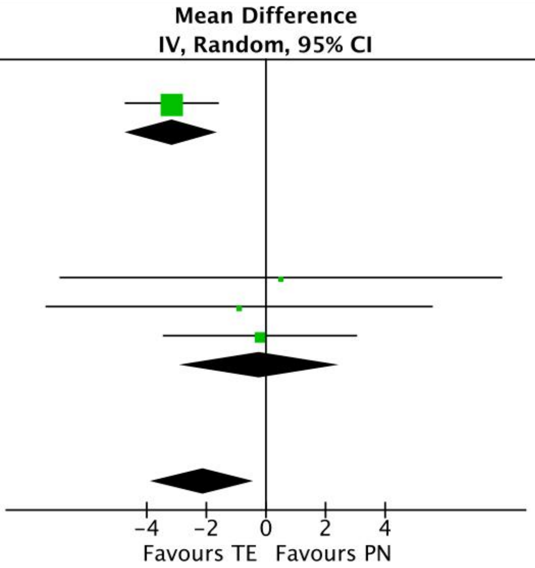

C

Study or Subgroup	TE Events	TE Total	PN Events	PN Total	Weight	Odds Ratio M-H, Random, 95% CI
**5.1.1 Robot Only**						
Blackwell 2016	0	57	3	53	2.1%	0.13 [0.01, 2.49]
Lei 2023	6	135	11	224	12.0%	0.90 [0.33, 2.49]
Lu 2019	1	94	1	72	2.4%	0.76 [0.05, 12.42]
Lu 2023	4	190	6	190	8.8%	0.66 [0.18, 2.38]
Minoda 2021	3	45	1	45	3.4%	3.14 [0.31, 31.42]
Takagi 2017	1	45	0	45	1.8%	3.07 [0.12, 77.32]
Zhao 2021	3	139	4	64	6.8%	0.33 [0.07, 1.52]
**Subtotal (95% CI)**		**705**		**693**	**37.2%**	**0.74 [0.39, 1.38]**
Total events	18		26			
Heterogeneity: $Tau^2 = 0.00$; $Chi^2 = 4.86$, df = 6 (P = 0.56); $I^2 = 0\%$						
Test for overall effect: Z = 0.95 (P = 0.34)						
**5.1.2 Other**						
Culpan 2021	62	848	13	222	19.9%	1.27 [0.68, 2.35]
Deng 2015	1	94	3	85	3.4%	0.29 [0.03, 2.88]
Dobrota 2020	1	29	2	54	3.0%	0.93 [0.08, 10.70]
Dong 2017	6	71	18	373	12.9%	1.82 [0.70, 4.76]
Longo 2014	2	147	10	145	6.7%	0.19 [0.04, 0.87]
Lu 2017	5	280	6	105	9.6%	0.30 [0.09, 1.00]
Mukkamala 2014	2	86	26	516	7.3%	0.45 [0.10, 1.93]
**Subtotal (95% CI)**		**1555**		**1500**	**62.8%**	**0.65 [0.33, 1.31]**
Total events	79		78			
Heterogeneity: $Tau^2 = 0.40$; $Chi^2 = 12.30$, df = 6 (P = 0.06); $I^2 = 51\%$						
Test for overall effect: Z = 1.19 (P = 0.23)						
**Total (95% CI)**		**2260**		**2193**	**100.0%**	**0.73 [0.46, 1.13]**
Total events	97		104			
Heterogeneity: $Tau^2 = 0.16$; $Chi^2 = 17.28$, df = 13 (P = 0.19); $I^2 = 25\%$						
Test for overall effect: Z = 1.41 (P = 0.16)						
Test for subgroup differences: $Chi^2 = 0.06$, df = 1 (P = 0.81), $I^2 = 0\%$						

Odds Ratio M-H, Random, 95% CI

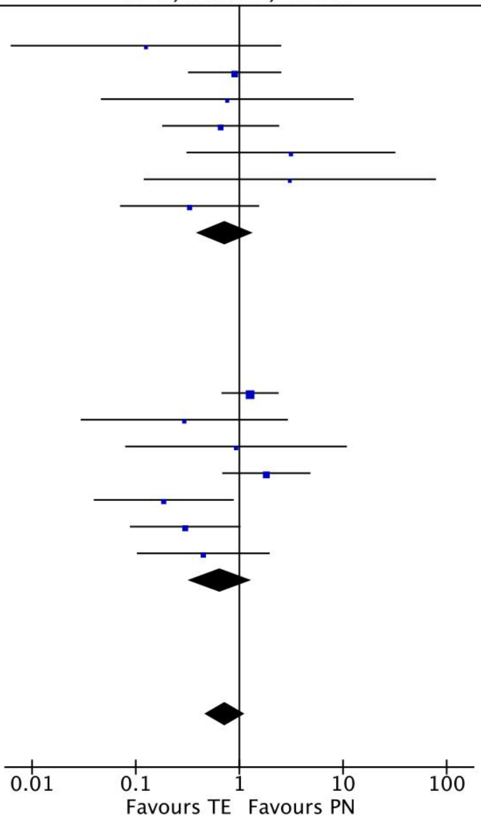

D

Study or Subgroup	Favours TE Events	Favours TE Total	PN Events	PN Total	Weight	Odds Ratio M-H, Random, 95% CI
**5.7.1 robot**						
Ellis 2024	2	168	16	252	7.2%	0.18 [0.04, 0.78]
Lei 2023	12	135	33	224	32.5%	0.56 [0.28, 1.14]
Lu 2019	0	94	2	72	1.7%	0.15 [0.01, 3.16]
Lu 2023	1	190	2	190	2.7%	0.50 [0.04, 5.53]
Minoda 2021	0	45	1	45	1.5%	0.33 [0.01, 8.22]
Takagi 2017	2	45	8	45	6.1%	0.22 [0.04, 1.08]
Zhao 2021	1	139	2	64	2.7%	0.22 [0.02, 2.52]
**Subtotal (95% CI)**		**816**		**892**	**54.5%**	**0.39 [0.23, 0.67]**
Total events	18		64			
Heterogeneity: $Tau^2 = 0.00$; $Chi^2 = 3.35$, df = 6 (P = 0.76); $I^2 = 0\%$						
Test for overall effect: Z = 3.42 (P = 0.0006)						
**5.7.2 other**						
Huang 2016	1	44	0	45	1.5%	3.14 [0.12, 79.13]
Longo 2014	10	198	9	198	18.6%	1.12 [0.44, 2.81]
Lu 2017	5	280	7	105	11.6%	0.25 [0.08, 0.82]
Mukkamala 2014	3	86	39	516	11.1%	0.44 [0.13, 1.46]
Wu 2020	1	90	2	93	2.7%	0.51 [0.05, 5.74]
**Subtotal (95% CI)**		**698**		**957**	**45.5%**	**0.59 [0.29, 1.20]**
Total events	20		57			
Heterogeneity: $Tau^2 = 0.14$; $Chi^2 = 5.08$, df = 4 (P = 0.28); $I^2 = 21\%$						
Test for overall effect: Z = 1.46 (P = 0.14)						
**Total (95% CI)**		**1514**		**1849**	**100.0%**	**0.48 [0.32, 0.71]**
Total events	38		121			
Heterogeneity: $Tau^2 = 0.00$; $Chi^2 = 9.57$, df = 11 (P = 0.57); $I^2 = 0\%$						
Test for overall effect: Z = 3.65 (P = 0.0003)						
Test for subgroup differences: $Chi^2 = 0.84$, df = 1 (P = 0.36), $I^2 = 0\%$						

Odds Ratio M-H, Random, 95% CI

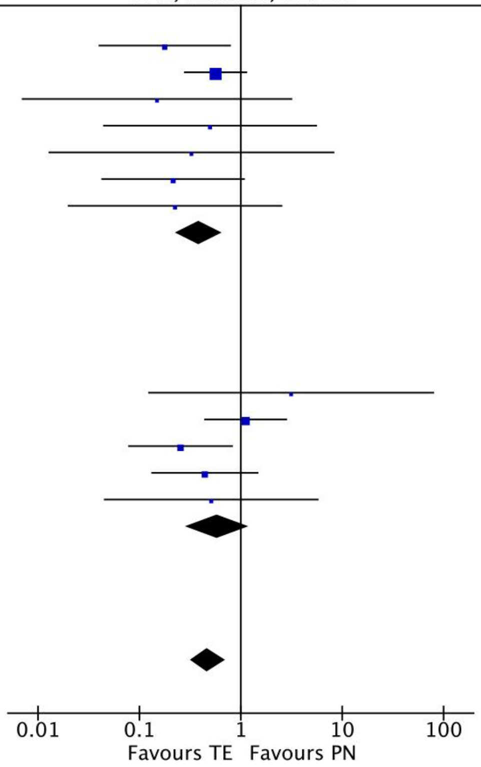

E

Study or Subgroup	TE Mean	TE SD	TE Total	PN Mean	PN SD	PN Total	Weight	Mean Difference IV, Random, 95% CI
**5.3.1 Robot Only**								
Blackwell 2016	24.33	6.67	57	25.5	5.93	53	7.5%	−1.17 [−3.53, 1.19]
Ellis 2024	10.7	10.1	168	23.4	11	252	7.8%	−12.70 [−14.74, −10.66]
Lei 2023	20.67	3.7	135	25.33	2.22	224	8.6%	−4.66 [−5.35, −3.97]
Lu 2019	17.5	3.7	94	20.5	3.5	72	8.4%	−3.00 [−4.10, −1.90]
Lu 2023	20.67	5.93	190	21.67	5.19	190	8.4%	−1.00 [−2.12, 0.12]
Minoda 2021	23	14	45	21	8	45	5.4%	2.00 [−2.71, 6.71]
Takagi 2017	24	12	45	20	6.4	45	6.0%	4.00 [0.03, 7.97]
Zhao 2021	21.2	6.4	139	24.1	6.9	64	7.8%	−2.90 [−4.90, −0.90]
**Subtotal (95% CI)**			**873**			**945**	**59.8%**	**−2.80 [−5.16, −0.44]**
Heterogeneity: Tau² = 10.09; Chi² = 130.91, df = 7 (P < 0.00001); I² = 95%								
Test for overall effect: Z = 2.33 (P = 0.02)								
**5.3.2 Other**								
Deng 2015	23.4	3.6	94	22.8	3.2	85	8.4%	0.60 [−0.40, 1.60]
Dong 2017	23	8.15	71	21.67	8.15	373	7.7%	1.33 [−0.74, 3.40]
Longo 2014	18	5.5	198	17.8	6.9	198	8.3%	0.20 [−1.03, 1.43]
Lu 2017	23.2	6.2	280	25.4	6.6	105	8.2%	−2.20 [−3.66, −0.74]
Mukkamala 2014	23	10	86	27	11	516	7.5%	−4.00 [−6.32, −1.68]
**Subtotal (95% CI)**			**729**			**1277**	**40.2%**	**−0.70 [−2.28, 0.88]**
Heterogeneity: Tau² = 2.57; Chi² = 22.49, df = 4 (P = 0.0002); I² = 82%								
Test for overall effect: Z = 0.87 (P = 0.39)								
**Total (95% CI)**			**1602**			**2222**	**100.0%**	**−2.00 [−3.76, −0.23]**
Heterogeneity: Tau² = 9.35; Chi² = 226.99, df = 12 (P < 0.00001); I² = 95%								
Test for overall effect: Z = 2.22 (P = 0.03)								
Test for subgroup differences: Chi² = 2.10, df = 1 (P = 0.15), I² = 52.4%								

Mean Difference IV, Random, 95% CI

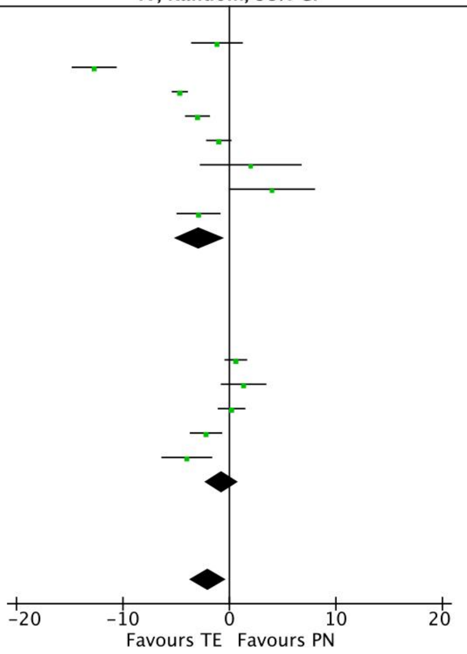

F

Study or Subgroup	TE Mean	TE SD	TE Total	PN Mean	PN SD	PN Total	Weight	Mean Difference IV, Random, 95% CI
**5.5.1 Robot Only**								
Lei 2023	165	22.2	135	173.3	18.5	224	9.7%	−8.30 [−12.76, −3.84]
Lu 2019	107.1	10.3	94	121.3	11.5	72	9.9%	−14.20 [−17.58, −10.82]
Lu 2023	145	37	190	156.3	41.5	190	8.8%	−11.30 [−19.21, −3.39]
Minoda 2021	140	44	45	167	40	45	5.8%	−27.00 [−44.37, −9.63]
Takagi 2017	189	37	45	193	43	45	6.0%	−4.00 [−20.57, 12.57]
Zhao 2021	197.7	54.6	139	215.6	61.6	64	5.7%	−17.90 [−35.51, −0.29]
**Subtotal (95% CI)**			**648**			**640**	**46.0%**	**−12.14 [−16.24, −8.04]**
Heterogeneity: Tau² = 9.01; Chi² = 8.47, df = 5 (P = 0.13); I² = 41%								
Test for overall effect: Z = 5.81 (P < 0.00001)								
**5.5.2 Other**								
Culpan 2021	151.7	51.9	848	130	66.7	222	8.3%	21.70 [12.26, 31.14]
Deng 2015	146	30.1	94	151.1	30.1	85	8.5%	−5.10 [−13.93, 3.73]
Huang 2016	100	38.8	44	124.8	42.8	45	5.9%	−24.80 [−41.76, −7.84]
Longo 2014	121	44	198	147	42	198	8.6%	−26.00 [−34.47, −17.53]
Lu 2017	182.1	52.6	280	192.8	53	105	7.5%	−10.70 [−22.56, 1.16]
Mukkamala 2014	159	58	86	191	58	516	7.1%	−32.00 [−45.24, −18.76]
Wu 2020	101.8	36.2	90	119.8	36.2	93	8.0%	−18.00 [−28.49, −7.51]
**Subtotal (95% CI)**			**1640**			**1264**	**54.0%**	**−13.21 [−27.41, 1.00]**
Heterogeneity: Tau² = 333.37; Chi² = 73.73, df = 6 (P < 0.00001); I² = 92%								
Test for overall effect: Z = 1.82 (P = 0.07)								
**Total (95% CI)**			**2288**			**1904**	**100.0%**	**−12.89 [−19.24, −6.54]**
Heterogeneity: Tau² = 102.84; Chi² = 82.48, df = 12 (P < 0.00001); I² = 85%								
Test for overall effect: Z = 3.98 (P < 0.0001)								
Test for subgroup differences: Chi² = 0.02, df = 1 (P = 0.89), I² = 0%								

Mean Difference IV, Random, 95% CI

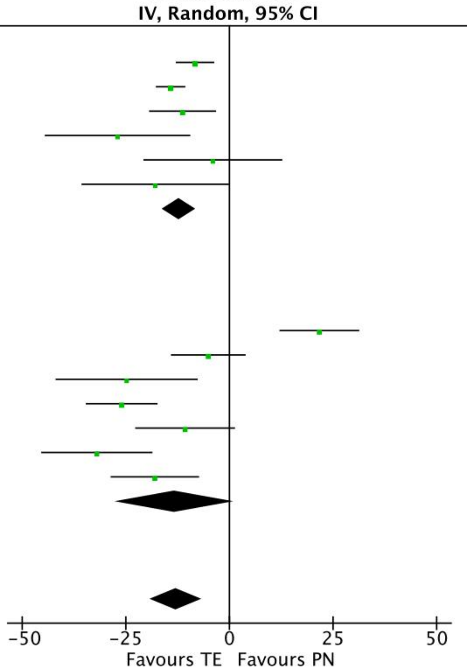

G

Study or Subgroup	TE Mean	TE SD	TE Total	PN Mean	PN SD	PN Total	Weight	Mean Difference IV, Random, 95% CI
**5.2.1 Robot Only**								
Ellis 2024	97.5	216.5	168	235.8	333.1	252	6.4%	−138.30 [−190.87, −85.73]
Lei 2023	126.7	44.4	135	155	48.1	224	8.4%	−28.30 [−38.09, −18.51]
Lu 2019	90.8	28.3	94	143.3	118.3	72	7.8%	−52.50 [−80.42, −24.58]
Lu 2023	116.7	111.1	190	100	74.1	190	8.1%	16.70 [−2.29, 35.69]
Minoda 2021	56	101	45	86	104	45	7.0%	−30.00 [−72.36, 12.36]
Takagi 2017	129	211	45	117	337	45	3.3%	12.00 [−104.17, 128.17]
Zhao 2021	230.5	207	139	269.8	273.3	64	5.1%	−39.30 [−114.58, 35.98]
**Subtotal (95% CI)**			**816**			**892**	**46.0%**	**−37.23 [−67.48, −6.97]**
Heterogeneity: Tau² = 1131.01; Chi² = 41.12, df = 6 (P < 0.00001); I² = 85%								
Test for overall effect: Z = 2.41 (P = 0.02)								
**5.2.2 Other**								
Culpan 2021	193.3	148.1	848	193.3	88.9	222	8.3%	0.00 [−15.37, 15.37]
Deng 2015	202.2	118	94	267.4	167.7	85	7.0%	−65.20 [−108.10, −22.30]
Dong 2017	56.7	59.3	71	200	148.1	373	8.1%	−143.30 [−163.70, −122.90]
Huang 2016	122.5	70	44	220	145	45	6.7%	−97.50 [−144.64, −50.36]
Longo 2014	177	128	198	221	131	198	7.9%	−44.00 [−69.51, −18.49]
Lu 2017	223.3	206.1	280	206.7	298.8	105	5.8%	16.60 [−45.44, 78.64]
Mukkamala 2014	359	620	86	371	562	516	2.6%	−12.00 [−151.72, 127.72]
Wu 2020	133.8	82.5	90	188.8	120	93	7.7%	−55.00 [−84.75, −25.25]
**Subtotal (95% CI)**			**1711**			**1637**	**54.0%**	**−53.72 [−101.14, −6.30]**
Heterogeneity: Tau² = 4023.84; Chi² = 130.31, df = 7 (P < 0.00001); I² = 95%								
Test for overall effect: Z = 2.22 (P = 0.03)								
**Total (95% CI)**			**2527**			**2529**	**100.0%**	**−47.34 [−74.14, −20.55]**
Heterogeneity: Tau² = 2202.83; Chi² = 189.09, df = 14 (P < 0.00001); I² = 93%								
Test for overall effect: Z = 3.46 (P = 0.0005)								
Test for subgroup differences: Chi² = 0.33, df = 1 (P = 0.57), I² = 0%								

Mean Difference IV, Random, 95% CI

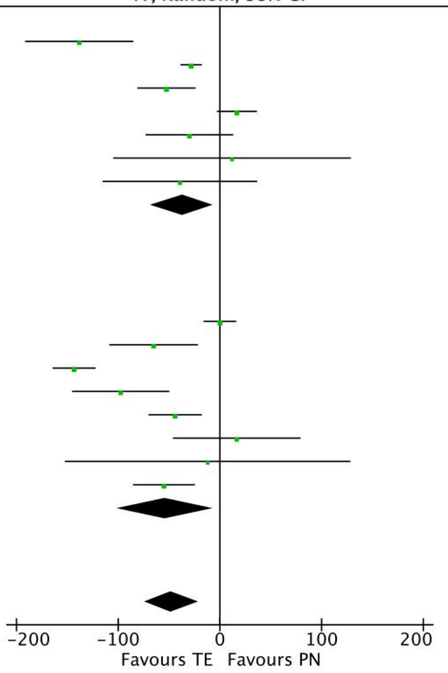

Figure S3. Subgroup analyses according to surgical platform.
(A) Postoperative eGFR. (B) Absolute ΔeGFR. (C) Positive surgical margin. (D) Major complications. (E) Warm ischemia time. (F) Operative time. (G) Estimated blood loss
