# Supplementary material for: Tumor enucleation versus conventional partial nephrectomy for localized renal tumors: a systematic review and meta-analysis of functional, perioperative, and margin outcomes
Source: Front Oncol. 2026 Jun 26;16:1853974. doi: 10.3389/fonc.2026.1853974 (PMC13349772; doi:10.3389/fonc.2026.1853974)
Supplement: Supplementary Figure 4 — Funnel plots for publication bias assessment. (A) Positive surgical margin. (B) Major complications. (C) Warm ischemia time. (D) Operative time. (E) Estimated blood loss. [file DataSheet4.pdf]

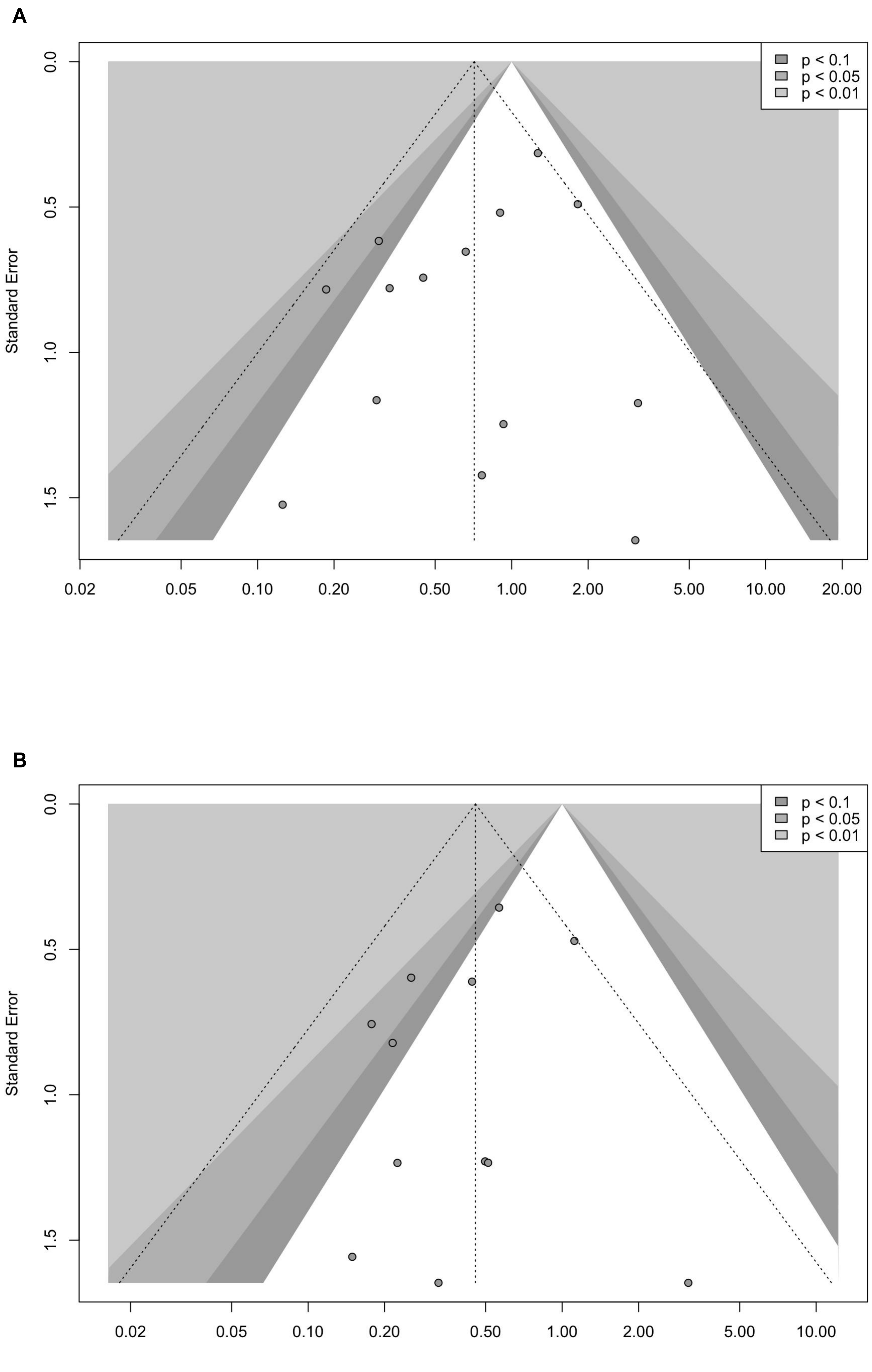
A
p < 0.1
p < 0.05
p < 0.01
Standard Error
0.0
0.5
1.0
1.5
0.02
0.05
0.10
0.20
0.50
1.00
2.00
5.00
10.00
20.00
B
p < 0.1
p < 0.05
p < 0.01
Standard Error
0.0
0.5
1.0
1.5
0.02
0.05
0.10
0.20
0.50
1.00
2.00
5.00
10.00

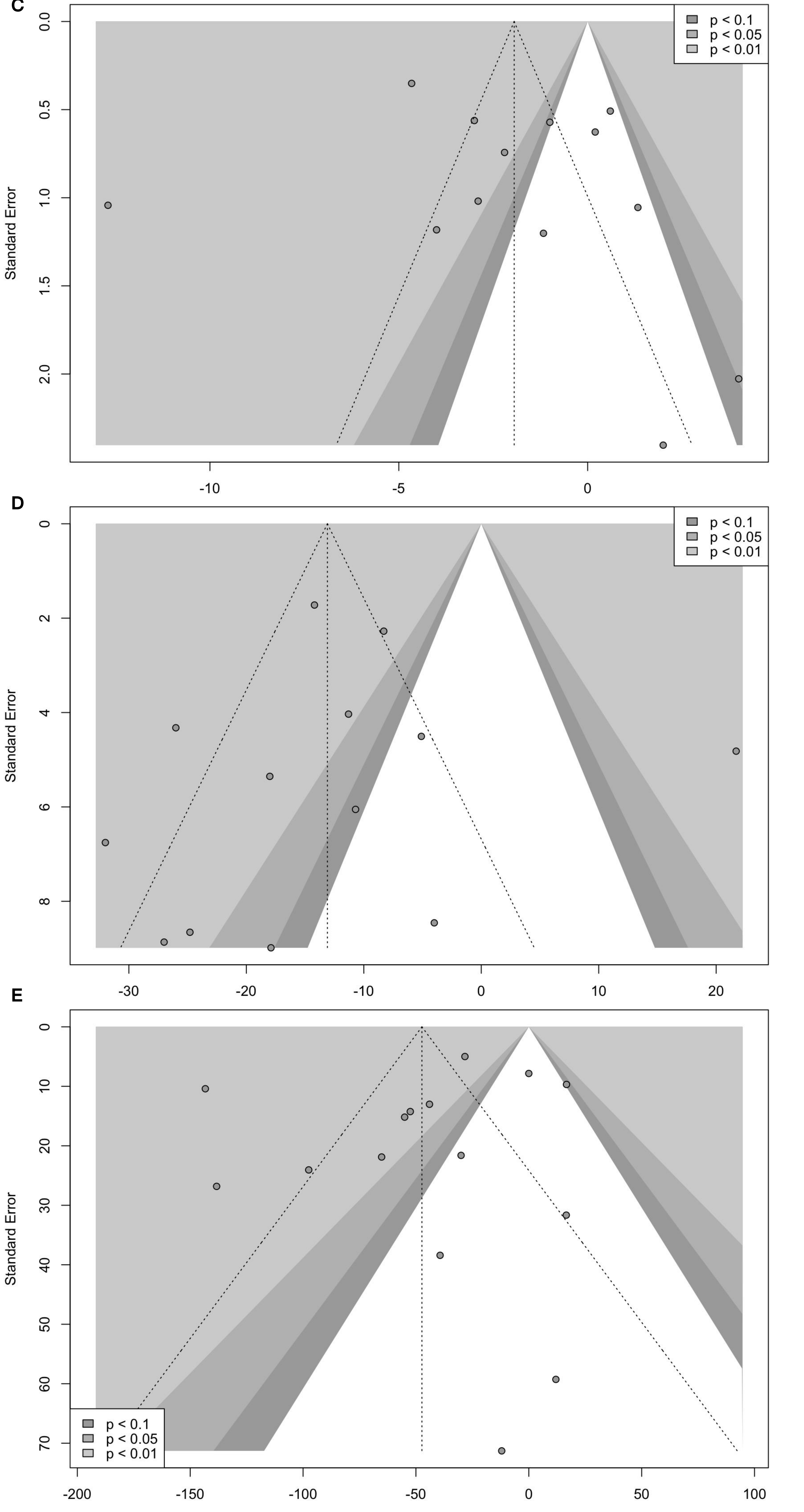
C
D
E
Standard Error
p < 0.1
p < 0.05
p < 0.01
0.0
0.5
1.0
1.5
2.0
-10
-5
0
0
2
4
6
8
-30
-20
-10
0
10
20
0
10
20
30
40
50
60
70
-200
-150
-100
-50
0
50
100

Figure S4. Funnel plots for publication bias assessment.
(A) Positive surgical margin. (B) Major complications. (C) Warm ischemia time. (D) Operative time. (E) Estimated blood loss
