# Supplementary material for: Tumor enucleation versus conventional partial nephrectomy for localized renal tumors: a systematic review and meta-analysis of functional, perioperative, and margin outcomes
Source: Front Oncol. 2026 Jun 26;16:1853974. doi: 10.3389/fonc.2026.1853974 (PMC13349772; doi:10.3389/fonc.2026.1853974)
Supplement: Supplementary Table 1 — Search strategies. [file Table1.docx]

**Supplementary Table S1. Search strategies**

*Note. No language or publication-type restrictions were applied during database searching. Reviews, case reports, conference abstracts, editorials, letters, and other non-original studies were excluded during title/abstract or full-text screening according to the eligibility criteria.*

**PubMed**

| 1. "Kidney Neoplasms"[Mesh]  2. "Carcinoma, Renal Cell"[Mesh]  3. renal tumor*[Title/Abstract]  4. renal tumour*[Title/Abstract]  5. kidney tumor*[Title/Abstract]  6. kidney tumour*[Title/Abstract]  7. renal mass*[Title/Abstract]  8. renal cell carcinoma[Title/Abstract]  9. renal carcinoma*[Title/Abstract]  10. kidney neoplasm*[Title/Abstract]  11. kidney cancer[Title/Abstract]  12. 1 OR 2 OR 3 OR 4 OR 5 OR 6 OR 7 OR 8 OR 9 OR 10 OR 11  13. partial nephrectom*[Title/Abstract]  14. subtotal nephrectom*[Title/Abstract]  15. nephron-sparing surgery[Title/Abstract]  16. nephron sparing surgery[Title/Abstract]  17. nephron-sparing resection[Title/Abstract]  18. kidney-sparing surgery[Title/Abstract]  19. conservative renal surgery[Title/Abstract]  20. conservative kidney surgery[Title/Abstract]  21. tumor enucleation[Title/Abstract]  22. tumour enucleation[Title/Abstract]  23. simple enucleation[Title/Abstract]  24. minimal margin enucleation[Title/Abstract]  25. enucleative resection[Title/Abstract]  26. enucleoresection[Title/Abstract]  27. 13 OR 14 OR 15 OR 16 OR 17 OR 18 OR 19 OR 20 OR 21 OR 22 OR 23 OR 24 OR 25 OR 26  28. 12 AND 27 |
| --- |

**Embase**

| 1. 'kidney tumor'/exp  2. 'renal cell carcinoma'/exp  3. 'kidney neoplasm'/exp  4. 'renal tumor*':ti,ab  5. 'renal tumour*':ti,ab  6. 'kidney tumor*':ti,ab  7. 'kidney tumour*':ti,ab  8. 'renal mass*':ti,ab  9. 'renal cell carcinoma':ti,ab  10. 'renal carcinoma*':ti,ab  11. 'kidney cancer':ti,ab  12. 1 OR 2 OR 3 OR 4 OR 5 OR 6 OR 7 OR 8 OR 9 OR 10 OR 11  13. 'partial nephrectomy'/exp  14. 'nephron sparing surgery'/exp  15. 'partial nephrectom*':ti,ab  16. 'subtotal nephrectom*':ti,ab  17. 'nephron-sparing surgery':ti,ab  18. 'nephron sparing surgery':ti,ab  19. 'nephron-sparing resection':ti,ab  20. 'kidney-sparing surgery':ti,ab  21. 'conservative renal surgery':ti,ab  22. 'conservative kidney surgery':ti,ab  23. 'tumor enucleation':ti,ab  24. 'tumour enucleation':ti,ab  25. 'simple enucleation':ti,ab  26. 'minimal margin enucleation':ti,ab  27. 'enucleative resection':ti,ab  28. 'enucleoresection':ti,ab  29. 13 OR 14 OR 15 OR 16 OR 17 OR 18 OR 19 OR 20 OR 21 OR 22 OR 23 OR 24 OR 25 OR 26 OR 27 OR 28  30. 12 AND 29 |
| --- |

**Web of Science**

| 1. TS=("renal tumor*" OR "renal tumour*" OR "kidney tumor*" OR "kidney tumour*" OR "renal mass*" OR "renal cell carcinoma" OR "renal carcinoma*" OR "kidney neoplasm*" OR "kidney cancer")  2. TS=("partial nephrectom*" OR "subtotal nephrectom*" OR "nephron-sparing surgery" OR "nephron sparing surgery" OR "nephron-sparing resection" OR "kidney-sparing surgery" OR "conservative renal surgery" OR "conservative kidney surgery" OR "tumor enucleation" OR "tumour enucleation" OR "simple enucleation" OR "minimal margin enucleation" OR "enucleative resection" OR "enucleoresection")  3. 1 AND 2 |
| --- |

**Cochrane Library**

| 1. MeSH descriptor: [Kidney Neoplasms] explode all trees  2. MeSH descriptor: [Carcinoma, Renal Cell] explode all trees  3. renal tumor*:ti,ab,kw  4. renal tumour*:ti,ab,kw  5. kidney tumor*:ti,ab,kw  6. kidney tumour*:ti,ab,kw  7. renal mass*:ti,ab,kw  8. renal cell carcinoma:ti,ab,kw  9. renal carcinoma*:ti,ab,kw  10. kidney neoplasm*:ti,ab,kw  11. kidney cancer:ti,ab,kw  12. 1 OR 2 OR 3 OR 4 OR 5 OR 6 OR 7 OR 8 OR 9 OR 10 OR 11  13. partial nephrectom*:ti,ab,kw  14. subtotal nephrectom*:ti,ab,kw  15. nephron-sparing surgery:ti,ab,kw  16. nephron sparing surgery:ti,ab,kw  17. nephron-sparing resection:ti,ab,kw  18. kidney-sparing surgery:ti,ab,kw  19. conservative renal surgery:ti,ab,kw  20. conservative kidney surgery:ti,ab,kw  21. tumor enucleation:ti,ab,kw  22. tumour enucleation:ti,ab,kw  23. simple enucleation:ti,ab,kw  24. minimal margin enucleation:ti,ab,kw  25. enucleative resection:ti,ab,kw  26. enucleoresection:ti,ab,kw  27. 13 OR 14 OR 15 OR 16 OR 17 OR 18 OR 19 OR 20 OR 21 OR 22 OR 23 OR 24 OR 25 OR 26  28. 12 AND 27 |
| --- |

**Scopus**

| 1. TITLE-ABS-KEY("renal tumor*" OR "renal tumour*" OR "kidney tumor*" OR "kidney tumour*" OR "renal mass*" OR "renal cell carcinoma" OR "renal carcinoma*" OR "kidney neoplasm*" OR "kidney cancer")  2. TITLE-ABS-KEY("partial nephrectom*" OR "subtotal nephrectom*" OR "nephron-sparing surgery" OR "nephron sparing surgery" OR "nephron-sparing resection" OR "kidney-sparing surgery" OR "conservative renal surgery" OR "conservative kidney surgery" OR "tumor enucleation" OR "tumour enucleation" OR "simple enucleation" OR "minimal margin enucleation" OR "enucleative resection" OR "enucleoresection")  3. 1 AND 2 |
| --- |

**CNKI**

| 1. 肾肿瘤  2. 肾癌  3. 肾细胞癌  4. 肾占位  5. 肾肿块  6. 肾实质肿瘤  7. 肾恶性肿瘤  8. 1 OR 2 OR 3 OR 4 OR 5 OR 6 OR 7  9. 部分肾切除  10. 肾部分切除  11. 肾次全切除  12. 保留肾单位  13. 保留肾单位手术  14. 肾单位保留手术  15. 保肾手术  16. 肿瘤剜除  17. 简单剜除  18. 剜除术  19. 9 OR 10 OR 11 OR 12 OR 13 OR 14 OR 15 OR 16 OR 17 OR 18  20. 8 AND 19 |
| --- |
