# Supplementary material for: Tumor enucleation versus conventional partial nephrectomy for localized renal tumors: a systematic review and meta-analysis of functional, perioperative, and margin outcomes
Source: Front Oncol. 2026 Jun 26;16:1853974. doi: 10.3389/fonc.2026.1853974 (PMC13349772; doi:10.3389/fonc.2026.1853974)
Supplement: Supplementary Table 2 — Technical Definitions of TE/PN and Zero-Ischemia Use. [file Table2.docx]

**Supplementary Table S2: Technical Definitions of TE/PN and Zero-Ischemia Use**

Abbreviations: TE, tumor enucleation; PN, partial nephrectomy; SPN, standard partial nephrectomy; LPN, laparoscopic partial nephrectomy; RAPN, robot-assisted partial nephrectomy; RATE, robot-assisted tumor enucleation;

**Supplementary Table S2A. Technical classification of TE and PN definitions across included studies**

| Study | TE definition category | PN comparator category |
| --- | --- | --- |
| Huang 2016 | Ablation-assisted zero-ischemia enucleation | Conventional laparoscopic PN |
| Lu 2023 | Clearly defined robot-assisted simple enucleation | Standard robot-assisted PN |
| Wu 2020 | Ablation-assisted zero-ischemia enucleation | Conventional laparoscopic PN |
| Longo 2014 | Clearly defined simple enucleation without visible parenchymal rim around tumor pseudocapsule | Standard PN with additional visible margin of healthy renal parenchyma |
| Minoda 2021 | SIB score-based enucleation | SIB score-based standard resection |
| Takagi 2017 | SIB score-based enucleation | SIB score-based standard resection |
| Blackwell 2016 | Clearly defined pseudocapsule-plane TE | Sharp standard PN with intentional parenchymal rim |
| Culpan 2021 | Clearly defined pseudocapsule-plane TE | Standard PN |
| Deng 2015 | Clearly defined pseudocapsule-plane enucleation | LPN with 0.5-1.0 cm margin from tumor edge |
| Dobrota 2020 | Clearly defined pseudocapsule-plane enucleation | Standard PN with safety margin |
| Dong 2017 | Clearly defined capsule/pseudocapsule-plane TE | SPN with intentional parenchymal rim |
| Ellis 2024 | Clearly defined pseudocapsule-plane TE | SPN with parenchymal rim |
| Lei 2023 | Clearly defined robotic pseudocapsule-plane TE | RAPN with removal of adjacent normal tissue |
| Lu 2017 | Clearly defined modified laparoscopic simple enucleation | Standard laparoscopic PN |
| Lu 2019 | Modified robot-assisted tumor enucleation | Conventional robot-assisted PN |
| Mukkamala 2014 | Clearly defined pseudocapsule-plane TE | Sharp excision |
| Zhao 2021 | Clearly defined modified robot-assisted simple enucleation | Standard robot-assisted PN |

Note: TE and PN definitions were broadly classified according to the operative principle described in each study.

**Supplementary Table S2B. Simplified grouping of TE/PN technical definitions**

| Category | Studies | Description |
| --- | --- | --- |
| Clearly defined pseudocapsule-plane or simple enucleation vs standard PN/sharp excision | Blackwell 2016; Culpan 2021; Deng 2015; Dobrota 2020; Dong 2017; Ellis 2024; Lei 2023; Longo 2014; Lu 2017; Lu 2023; Mukkamala 2014; Zhao 2021 | Most studies used tumor removal along the pseudocapsule or natural plane without intentional removal of a visible rim of normal parenchyma. |
| Ablation-assisted zero-ischemia enucleation vs conventional LPN | Huang 2016; Wu 2020 | TE was combined with radiofrequency or microwave ablation and was designed as a zero-ischemia/no-clamping approach. |
| SIB score-based enucleation vs standard resection | Minoda 2021; Takagi 2017 | The resection technique was classified according to the Surface-Intermediate-Base margin score. |
| Modified robot-assisted enucleation for complex tumors | Lu 2019 | Modified robot-assisted enucleation was used for highly complex renal tumors. |

**Supplementary Table S3C. Use of zero-ischemia or no-clamping techniques across included studies**

| Category | Studies | Description |
| --- | --- | --- |
| Full zero-ischemia TE technique | Huang 2016; Wu 2020 | The TE arm was designed as zero-ischemia ablation-assisted enucleation without hilar clamping. |
| Partial zero-ischemia / no-clamping use | Dong 2017; Lu 2017 | Some patients underwent TE or NSS without hilar clamping, but not all cases used zero-ischemia. |
| Surgeon-selected warm ischemia or no ischemia | Blackwell 2016; Dobrota 2020 | Use of clamping depended on surgeon preference, bleeding, tumor location, or intraoperative findings. |
| Not clearly reported as zero-ischemia | Lu 2023; Longo 2014; Minoda 2021; Takagi 2017; Culpan 2021; Deng 2015; Ellis 2024; Lei 2023; Lu 2019; Mukkamala 2014; Zhao 2021 | The original reports did not clearly define the TE arm as a zero-ischemia or no-clamping technique in the available extracted text. |
