# Supplementary material for: Tumor enucleation versus conventional partial nephrectomy for localized renal tumors: a systematic review and meta-analysis of functional, perioperative, and margin outcomes
Source: Front Oncol. 2026 Jun 26;16:1853974. doi: 10.3389/fonc.2026.1853974 (PMC13349772; doi:10.3389/fonc.2026.1853974)
Supplement: Supplementary Table 3 — Risk of bias assessment for randomized trials and Newcastle-Ottawa Scale assessment for non-randomized studies. [file Table3.docx]

**Supplementary Table S3. Risk of bias assessment for randomized trials and Newcastle-Ottawa Scale assessment for non-randomized studies**

**Supplementary Table S3A. RoB 2 assessment for randomized studies**

| Study | Randomization process | Deviations from intended interventions | Missing outcome data | Measurement of outcome | Selection of reported result | Overall risk |
| --- | --- | --- | --- | --- | --- | --- |
| Huang 2016 | Low risk | Some concerns | Some concerns | Some concerns | Low risk | Some concerns |
| Wu 2020 | Low risk | Some concerns | Some concerns | Some concerns | Low risk | Some concerns |
| Lu 2023 | Low risk | Low risk | Low risk | Low risk | Low risk | Low risk |

**Supplementary Table S3B. NOS assessment for observational studies**

| **Study** | **Selection** | **Comparability** | **Outcome** | **Total NOS score** | **Quality judgment** |
| --- | --- | --- | --- | --- | --- |
| Blackwell 2016 | 4 | 1 | 2 | 7 | High |
| Culpan 2021 | 4 | 2 | 2 | 8 | High |
| Dobrota 2020 | 4 | 1 | 2 | 7 | High |
| Dong 2017 | 4 | 2 | 2 | 8 | High |
| Ellis 2024 | 4 | 2 | 2 | 8 | High |
| Lei 2023 | 4 | 1 | 2 | 7 | High |
| Lu 2016 | 4 | 1 | 2 | 7 | High |
| Lu 2019 | 4 | 1 | 2 | 7 | High |
| Minoda 2021 | 4 | 2 | 2 | 8 | High |
| Mukkamala 2014 | 4 | 1 | 2 | 7 | High |
| Takagi 2017 | 4 | 2 | 2 | 8 | High |
| Zhao 2021 | 4 | 1 | 2 | 7 | High |
| Deng 2015 | 4 | 1 | 2 | 7 | High |
| Longo 2014 | 4 | 2 | 2 | 8 | High |

**Abbreviations:** NOS, Newcastle-Ottawa Scale; RoB 2, Cochrane Risk of Bias 2 tool.

**Note:** For the NOS assessment, scores of 7-9 were considered high quality. For RoB 2, each randomized trial was assessed across five domains, and the overall judgment was assigned according to the highest level of concern across domains.
