# Supplementary material for: Tumor enucleation versus conventional partial nephrectomy for localized renal tumors: a systematic review and meta-analysis of functional, perioperative, and margin outcomes
Source: Front Oncol. 2026 Jun 26;16:1853974. doi: 10.3389/fonc.2026.1853974 (PMC13349772; doi:10.3389/fonc.2026.1853974)
Supplement: Supplementary Table 4 — Univariable meta-regression analyses for perioperative outcomes. [file Table4.docx]

**Supplementary Table S4. Univariable meta-regression analyses for perioperative outcomes.**

| Outcome | Covariate | No. of studies | Coefficient | SE | 95% CI | P value | Tau² | Residual I² (%) |
| --- | --- | --- | --- | --- | --- | --- | --- | --- |
| WIT | Robot-assisted surgery | 11 | -2.22 | 2.86 | -7.83 to 3.39 | 0.438 | 17.14 | 93.62 |
| WIT | High-complexity tumors | 13 | 1.06 | 2.4 | -3.65 to 5.78 | 0.658 | 16.24 | 93.95 |
| WIT | Mean tumor size | 13 | -0.99 | 1.58 | -4.09 to 2.11 | 0.531 | 15.81 | 93.68 |
| WIT | Publication year | 13 | -0.58 | 0.28 | -1.14 to -0.02 | 0.041 | 11.18 | 91.44 |
| Operative time | Robot-assisted surgery | 12 | 8.69 | 7.68 | -6.36 to 23.73 | 0.258 | 138.13 | 85.29 |
| Operative time | High-complexity tumors | 13 | -1.07 | 8.35 | -17.44 to 15.3 | 0.898 | 176.85 | 86.61 |
| Operative time | Mean tumor size | 13 | 4.91 | 5.33 | -5.54 to 15.36 | 0.357 | 164.23 | 86.49 |
| Operative time | Ablation-assisted zero-ischemia TE | 13 | -9.26 | 11.17 | -31.15 to 12.63 | 0.407 | 164.38 | 86.11 |
| Operative time | Publication year | 13 | 1.58 | 1.18 | -0.74 to 3.89 | 0.183 | 146.53 | 83.95 |
| EBL | Complete zero-ischemia strategy | 15 | -32.19 | 39.49 | -109.6 to 45.22 | 0.415 | 2277.44 | 92.89 |
| EBL | Robot-assisted surgery | 13 | 19.79 | 24.43 | -28.09 to 67.67 | 0.418 | 1374.16 | 81.06 |
| EBL | High-complexity tumors | 15 | 22.14 | 29.94 | -36.54 to 80.82 | 0.46 | 2297.32 | 93.04 |
| EBL | Mean tumor size | 15 | 17.39 | 19.39 | -20.61 to 55.4 | 0.37 | 2218.19 | 92.75 |
| EBL | Publication year | 15 | 2.14 | 4.34 | -6.38 to 10.65 | 0.623 | 2265.24 | 90.61 |

Note: Univariable meta-regression was performed for perioperative outcomes with high heterogeneity. Coefficients represent study-level associations between each covariate and the pooled effect estimate.

Abbreviations: EBL, estimated blood loss; SE, standard error; TE, tumor enucleation; WIT, warm ischemia time; CI, confidence interval; Tau², between-study variance; I², inconsistency index.
